# Supplementary material for: The utility of serology for elimination surveillance of trachoma
Source: Nat Commun. 2018 Dec 21;9:5444. doi: 10.1038/s41467-018-07852-0 (PMC6303365; doi:10.1038/s41467-018-07852-0)
Supplement: Supplementary file 1 — Supplementary Information [file 41467_2018_7852_MOESM1_ESM.pdf]

The utility of serology for elimination surveillance of trachoma

Pinsent et al.

Supplementary Information file: Mathematical details of the antibody acquisition and sero-catalytic models, Tables of estimated parameter values, and supplementary figures of data and all of the transmission scenario fits to all 9 datasets.

Supplementary Figures: Figures of the individual-level antibody data for each site and the fits of all sero-catalytic and antibody acquisition to each study site

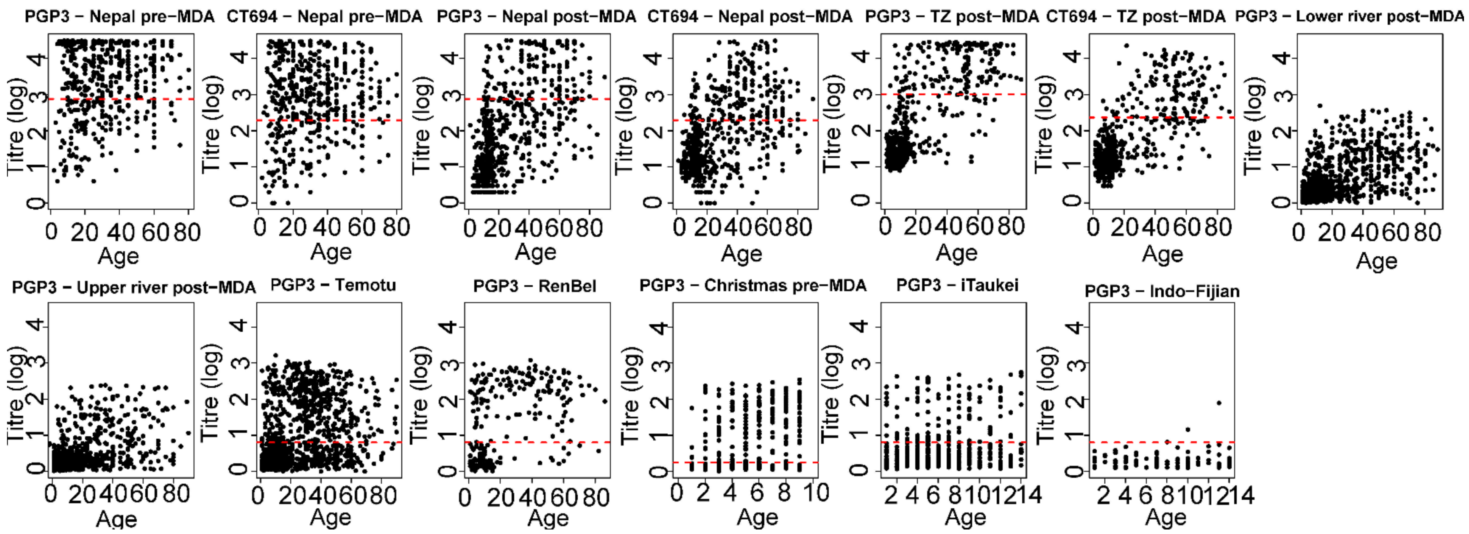

Supplementary Figure 1: For each dataset we plot age against antibody titre for each participant. We see an overall trend of increasing antibody titre with increasing age. In the post-MDA data for both antigens we see the average titre is lower, particularly in the younger age groups. Age-specific increases in antibody titre we much less pronounced in data from children in Fiji and Kiribati. Red dashed lines on each figure indicate the cut-off values used to define seropositive and negative individuals.

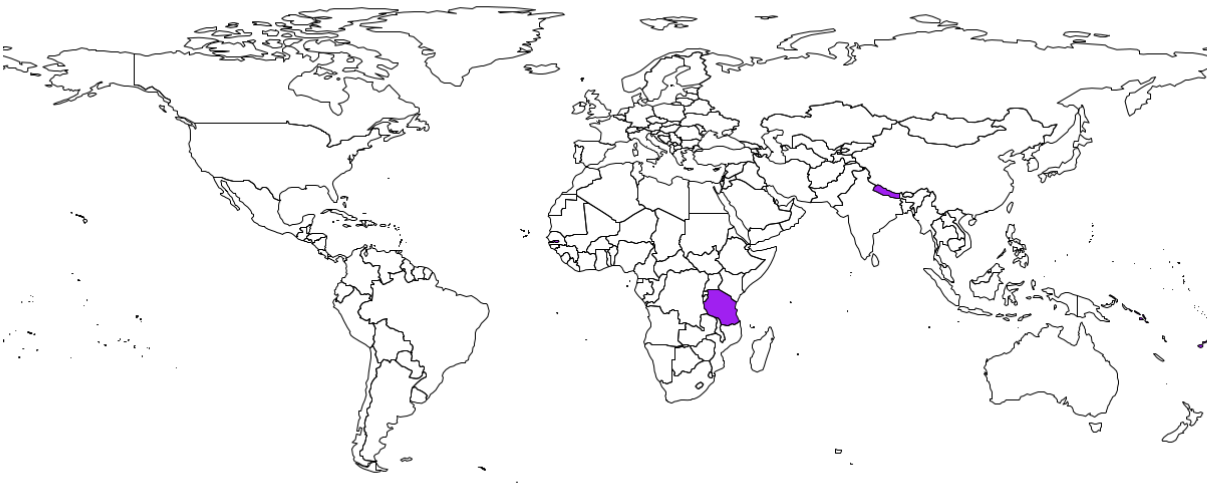

Supplementary Figure 2: A map of the world indicating in purple countries for which data was available and was included in our analysis. The figure was generated using R (1) using a freely available open source map template available within the package 'rworldmaps' (2).

Fits of each sero-catalytic models to each individual dataset

In Supplementary Figures 3-13 we present the fit of each model evaluated to each dataset separately. Parameter estimates for each model for each site are presented.

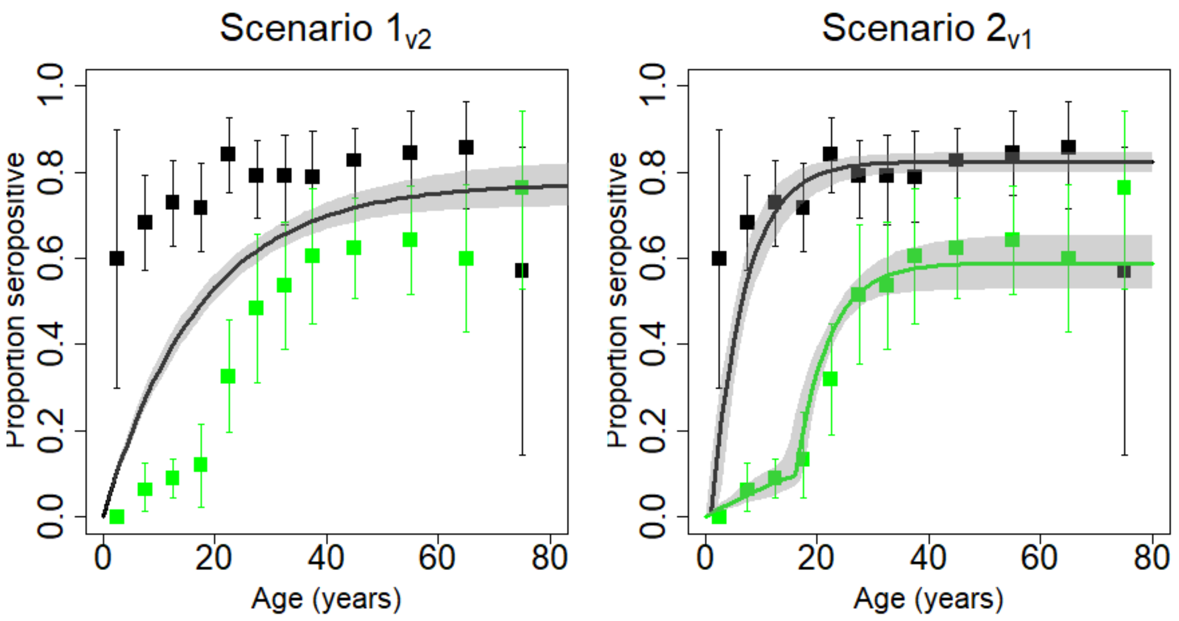

Supplementary Figure 3: Fits of each sero-catalytic model to age-specific sero-prevalence data from pre and post-MDA cross-sections from Nepal for PGP3. Data presented in black represent the pre-MDA data and data in green represent post-MDA data. Each square within each panel represents the age-group-specific sero-prevalence, the lines above and below each square indicate the 95% binomial confidence intervals associated with the reported age-group specific prevalence. The titles within each panel report the transmission scenario from which the parameter estimates and fits have been obtained. Solid black lines were generated using the median parameter estimates from each model fit. Solid black lines running through the sero-prevalence data were generated with the median parameter estimates from each model fit. The shaded grey regions about the black line represent 95% credible intervals of the model predictions. Uncertainty was generated by drawing 500 independent samples from the posterior distribution and re-running the model with these new values, this was done to understand variation in the model prediction.

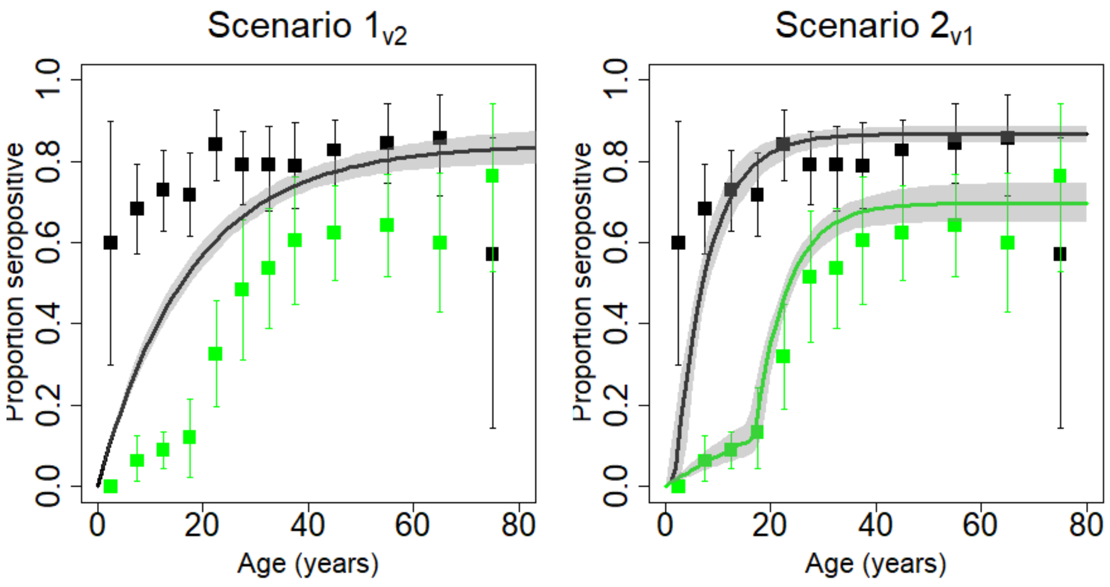

Supplementary Figure 4: Fits of each sero-catalytic model to age-specific sero-prevalence data from the cross-sections from Nepal for CT694. Data presented in black represent the pre-MDA data and data in green represent post-MDA data. Each square within each panel represents the age-group-specific sero-prevalence, the lines above and below each square indicate the 95% binomial confidence intervals associated with the reported age-group specific prevalence. The titles within each panel report the transmission scenario from which the parameter estimates and fits have been obtained. Solid black lines were generated using the median parameter estimates from each model fit. Solid black lines running through the sero-prevalence data were generated with the median parameter estimates from each model fit. The shaded grey regions about the black line represent 95% credible intervals of the model predictions. Uncertainty was generated by drawing 500 independent samples from the posterior distribution and re-running the model with these new values, this was done to understand variation in the model prediction.

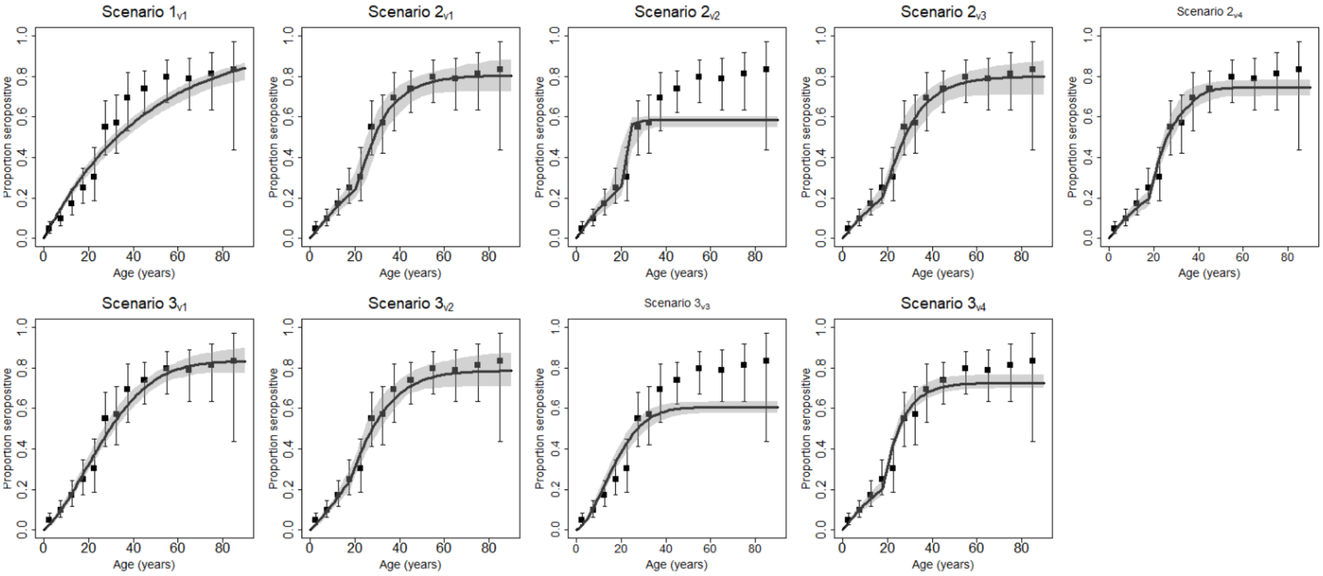

Supplementary Figure 5: Fits of each sero-catalytic model to age-specific sero-prevalence data from The Gambia, Lower River Region for PGP3. Each square within each panel represents the age-group-specific sero-prevalence, the lines above and below each square indicate the 95% binomial confidence intervals associated with the reported age-group specific prevalence. The titles within each panel report the transmission scenario from which the parameter estimates and fits have been obtained. Solid black lines were generated using the median parameter estimates from each model fit. Solid black lines running through the sero-prevalence data were generated with the median parameter estimates from each model fit. The shaded grey regions about the black line represent 95% credible intervals of the model predictions. Uncertainty was generated by drawing 500 independent samples from the posterior distribution and re-running the model with these new values, this was done to understand variation in the model prediction.

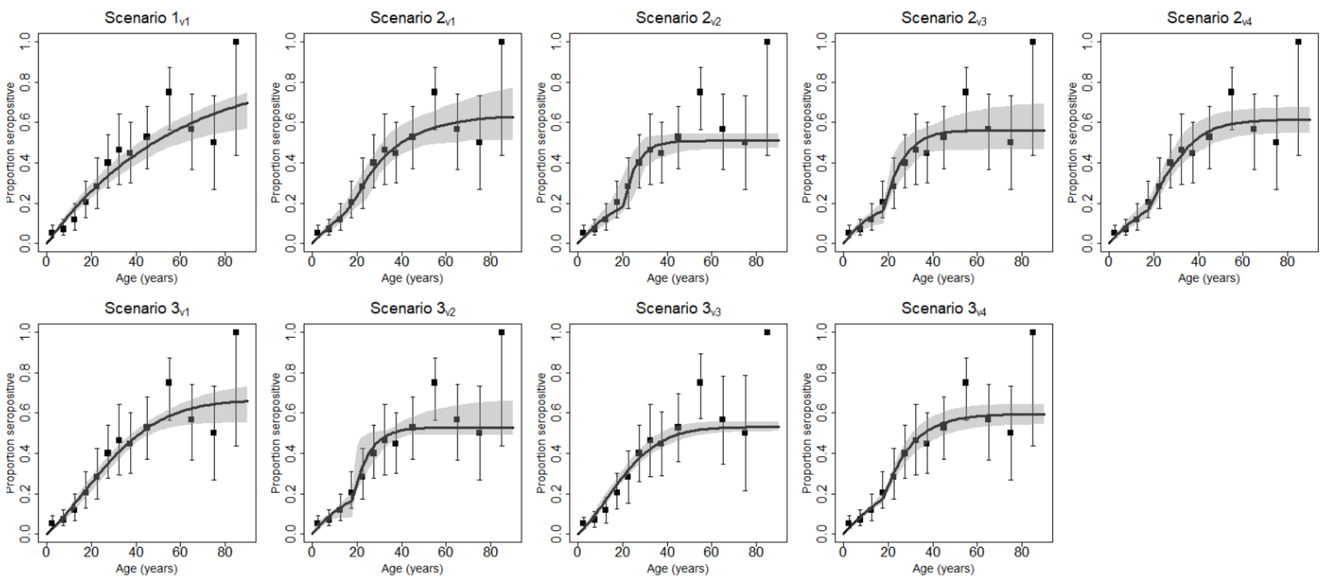

Supplementary Figure 6: Fits of each sero-catalytic model to age-specific sero-prevalence data from The Gambia, Upper River Region for PGP3. Each square within each panel represents the age-group-specific sero-prevalence, the lines above and below each square indicate the 95% binomial confidence intervals associated with the reported age-group specific prevalence. The titles within each panel report the transmission scenario from which the parameter estimates and fits have been obtained. Solid black lines were generated using the median parameter estimates from each model fit. Solid black lines running through the sero-prevalence data were generated with the median parameter estimates from each model fit. The shaded grey regions about the black line represent 95% credible intervals of the model predictions. Uncertainty was generated by drawing 500 independent samples from the posterior distribution and re-running the model with these new values, this was done to understand variation in the model prediction.

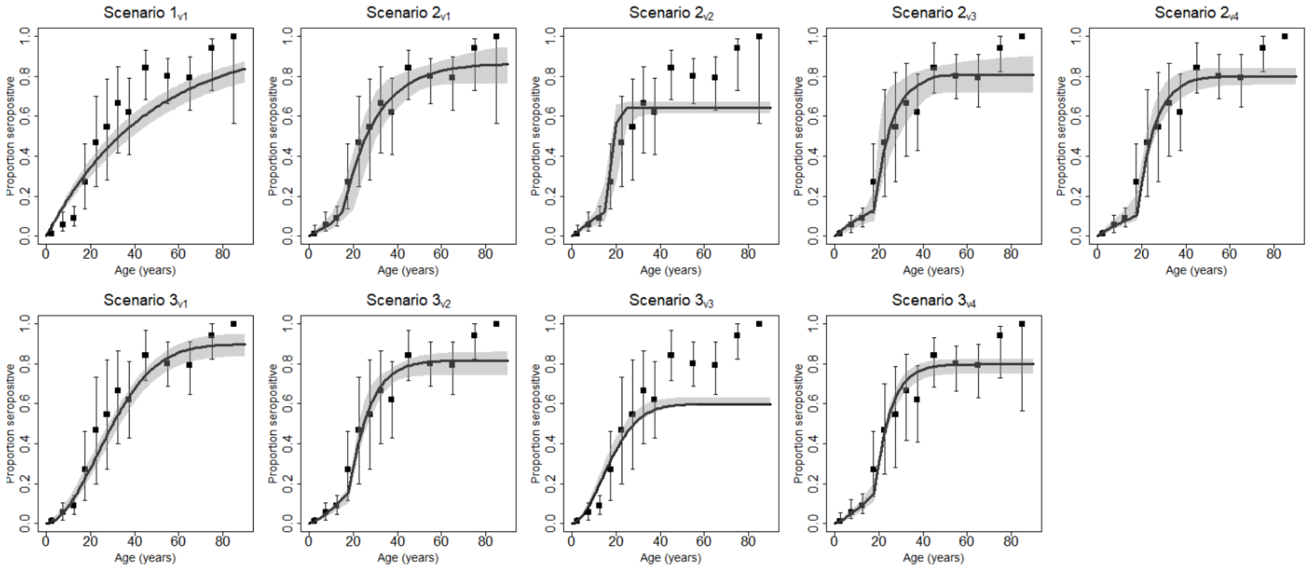

Supplementary Figure 7: Fits of each sero-catalytic model to age-specific sero-prevalence data from Tanzania, Rombo district for PGP3. Each square within each panel represents the age-group-specific sero-prevalence, the lines above and below each square indicate the 95% binomial confidence intervals associated with the reported age-group specific prevalence. The titles within each panel report the transmission scenario from which the parameter estimates and fits have been obtained. Solid black lines were generated using the median parameter estimates from each model fit. Solid black lines running through the sero-prevalence data were generated with the median parameter estimates from each model fit. The shaded grey regions about the black line represent 95% credible intervals of the model predictions. Uncertainty was generated by drawing 500

independent samples from the posterior distribution and re-running the model with these new values, this was done to understand variation in the model prediction.

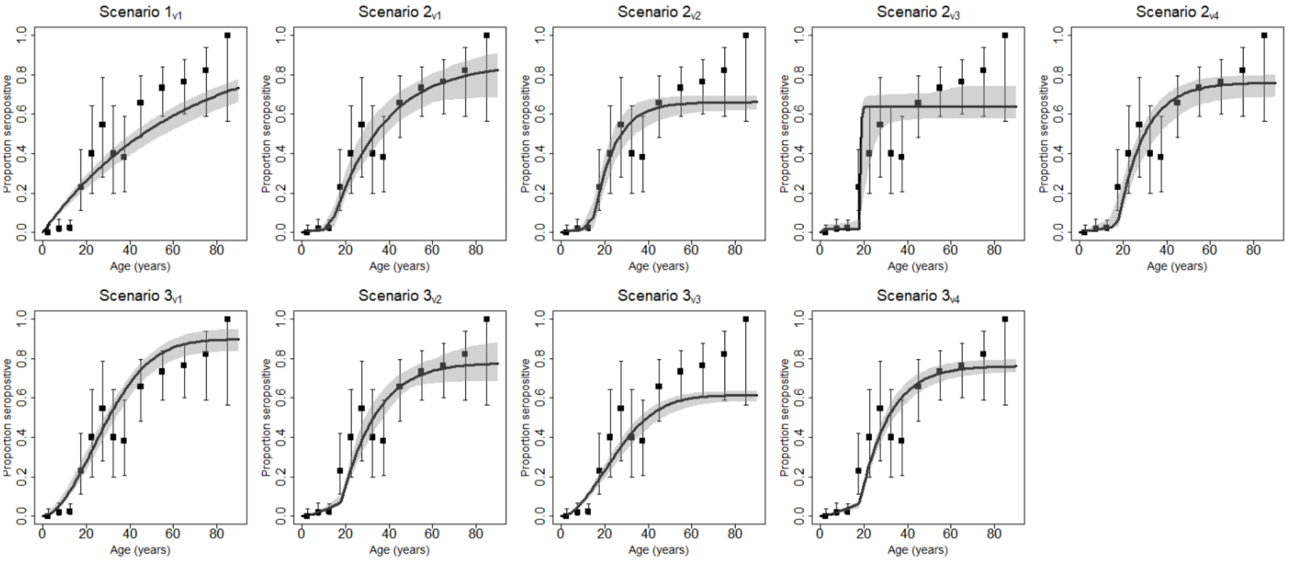

Supplementary Figure 8: Fits of each sero-catalytic model to age-specific sero-prevalence data from Tanzania, Rombo district for CT694. Each square within each panel represents the age-group-specific sero-prevalence, the lines above and below each square indicate the 95% binomial confidence intervals associated with the reported age-group specific prevalence. The titles within each panel report the transmission scenario from which the parameter estimates and fits have been obtained. Solid black lines were generated using the median parameter estimates from each model fit. Solid black lines running through the sero-prevalence data were generated with the median parameter estimates from each model fit. The shaded grey regions about the black line represent 95% credible intervals of the model predictions. Uncertainty was generated by drawing 500 independent samples from the posterior distribution and re-running the model with these new values, this was done to understand variation in the model prediction.

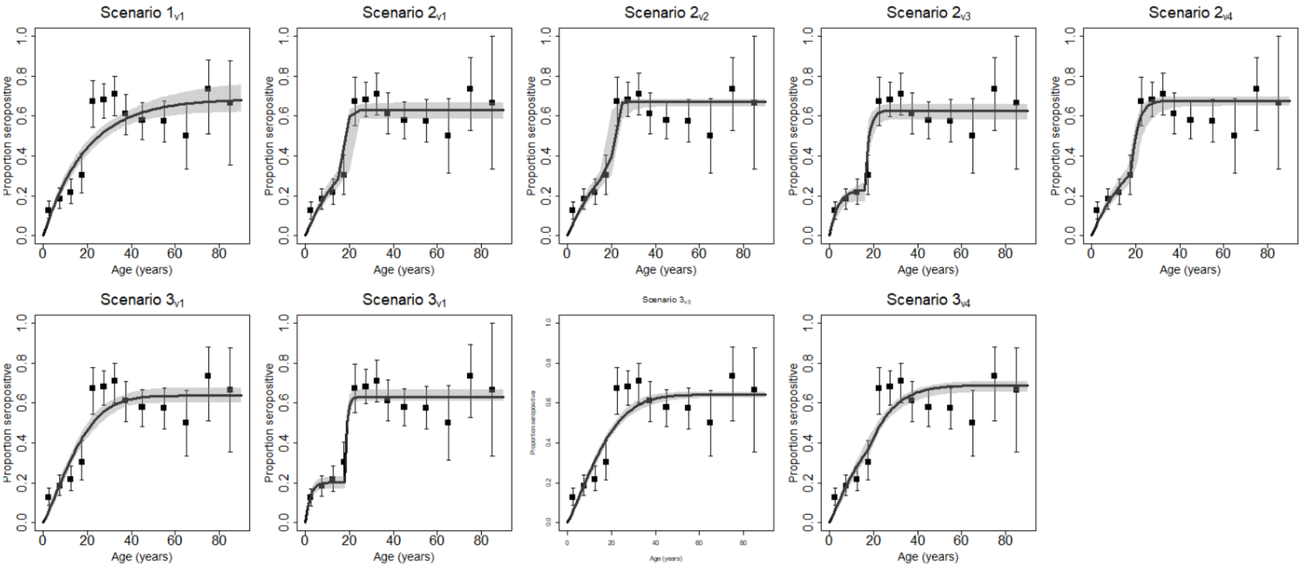

Supplementary Figure 9: Fits of each sero-prevalence model to age-specific sero-prevalence data from The Solomon Islands, Temotu district for PGP3. Each square within each panel represents the age-group-specific sero-prevalence, the lines above and below each square indicate the 95% binomial confidence intervals associated with the reported age-group specific prevalence. The titles within each panel report the transmission scenario from which the parameter estimates and fits have been obtained. Solid black lines were generated using the median parameter estimates from each model fit. Solid black lines running through the sero-prevalence data were generated with the median parameter estimates from each model fit. The shaded grey regions about the

black line represent 95% credible intervals of the model predictions. Uncertainty was generated by drawing 500 independent samples from the posterior distribution and re-running the model with these new values, this was done to understand variation in the model prediction.

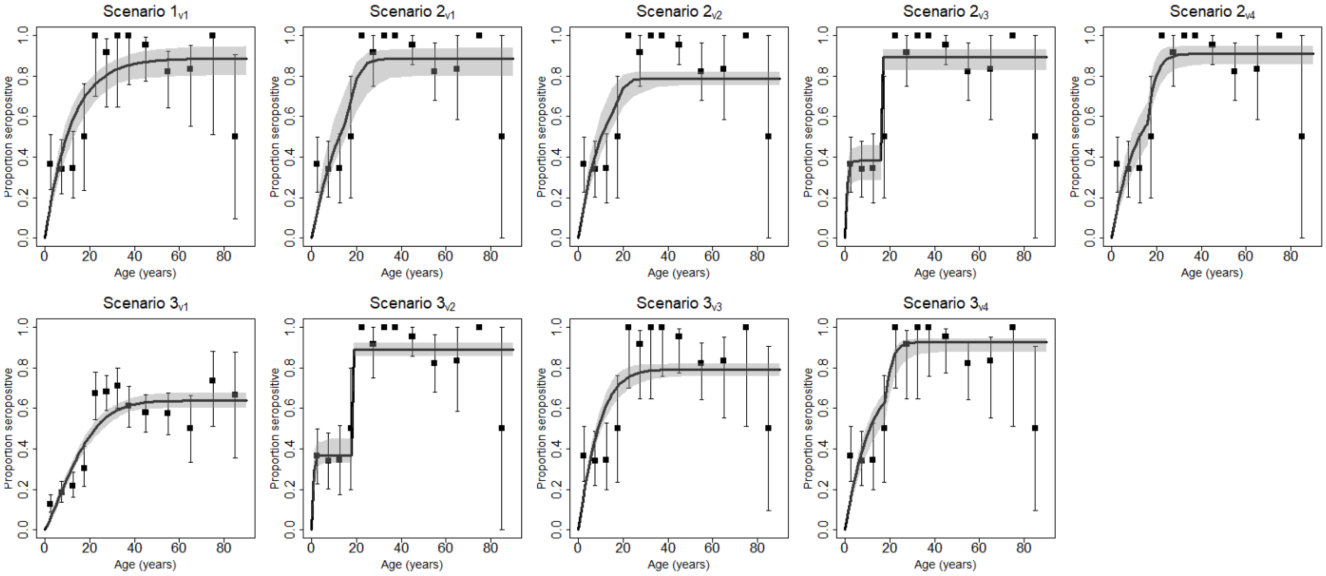

Supplementary Figure 10: Fits of each sero-catalytic model to age-specific sero-prevalence data from The Solomon Islands, RenBel district for PGP3. Each square within each panel represents the age-group-specific sero-prevalence, the lines above and below each square indicate the 95% binomial confidence intervals associated with the reported age-group specific prevalence. The titles within each panel report the transmission scenario from which the parameter estimates and fits have been obtained. Solid black lines were generated using the median parameter estimates from each model fit. Solid black lines running through the sero-prevalence data were generated with the median parameter estimates from each model fit. The shaded grey regions about the black line represent 95% credible intervals of the model predictions. Uncertainty was generated by drawing 500 independent samples from the posterior distribution and re-running the model with these new values, this was done to understand variation in the model prediction.

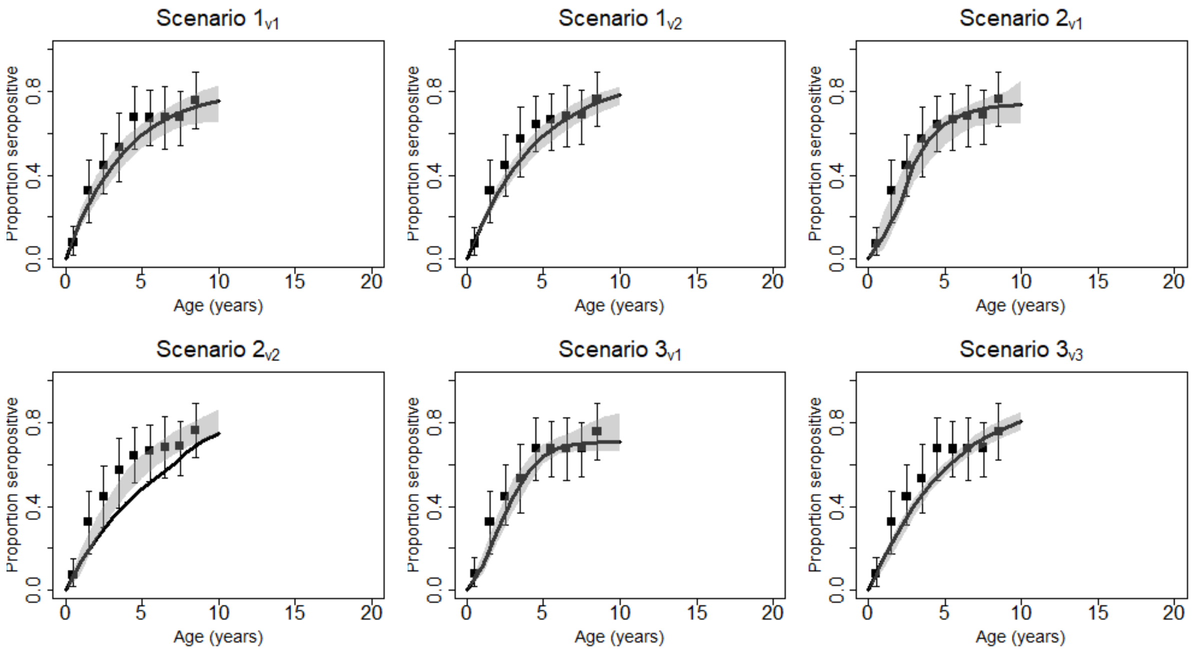

Supplementary Figure 11: Fits of each sero-catalytic model to age-specific sero-prevalence data from Kiribati for PGP3. Each square within each panel represents the age-group-specific sero-prevalence, the lines above and below each square indicate the 95% binomial confidence intervals associated with the reported age-group specific prevalence. The titles within each panel report the transmission scenario from which the

parameter estimates and fits have been obtained. Solid black lines were generated using the median parameter estimates from each model fit. Solid black lines running through the sero-prevalence data were generated with the median parameter estimates from each model fit. The shaded grey regions about the black line represent 95% credible intervals of the model predictions. Uncertainty was generated by drawing 500 independent samples from the posterior distribution and re-running the model with these new values, this was done to understand variation in the model prediction.

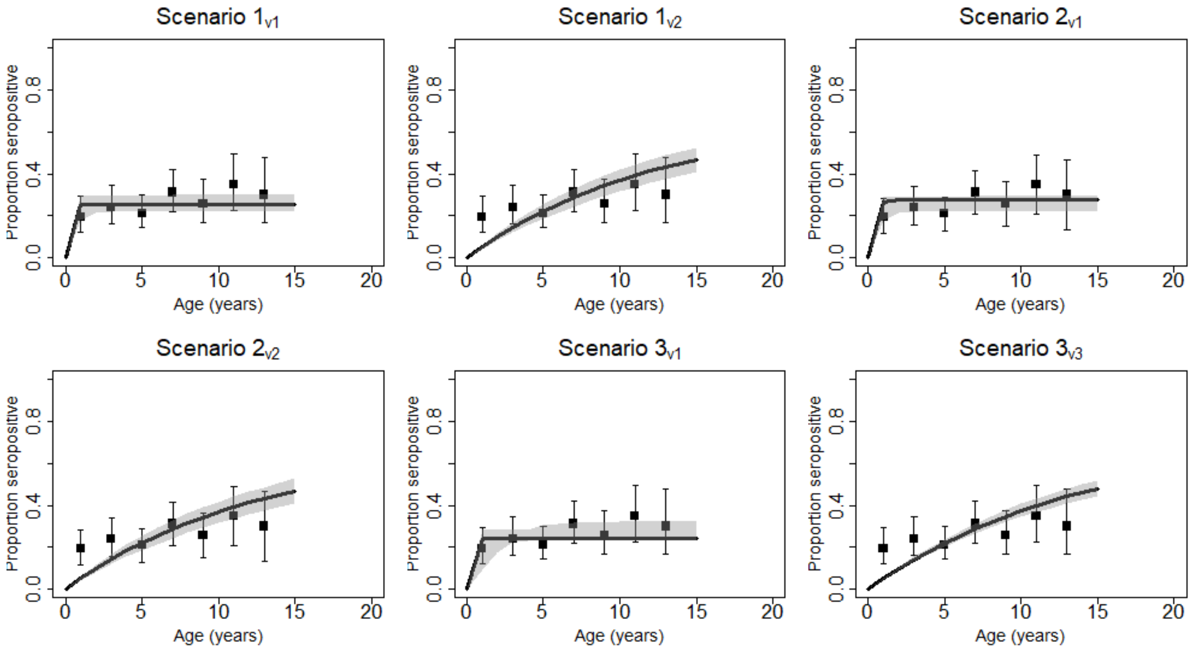

Supplementary Figure 12: Fits of each sero-catalytic model to age-specific sero-prevalence data from Fiji, from the iTaukei population for PGP3. Each square within each panel represents the age-group-specific sero-prevalence, the lines above and below each square indicate the 95% binomial confidence intervals associated with the reported age-group specific prevalence. The titles within each panel report the transmission scenario from which the parameter estimates and fits have been obtained. Solid black lines were generated using the median parameter estimates from each model fit. Solid black lines running through the sero-prevalence data were generated with the median parameter estimates from each model fit. The shaded grey regions about the black line represent 95% credible intervals of the model predictions. Uncertainty was generated by drawing 500 independent samples from the posterior distribution and re-running the model with these new values, this was done to understand variation in the model prediction.

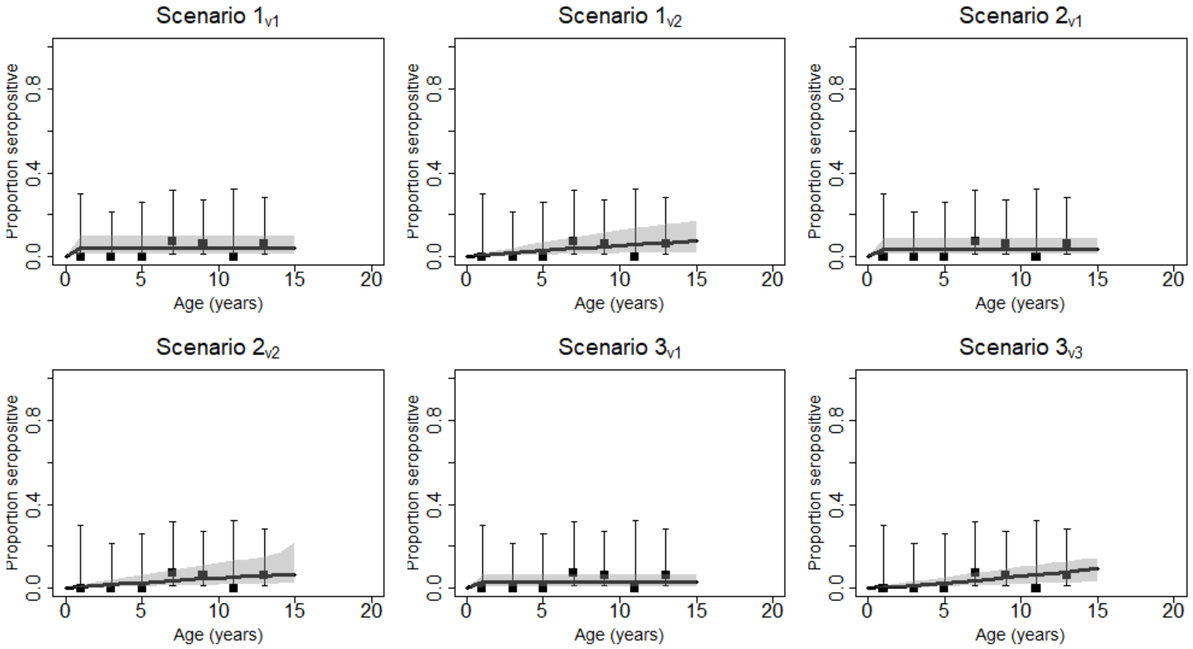

Supplementary Figure 13: Fits of each sero-catalytic model to age-specific sero-prevalence data from Fiji, from the Indo-Fijian population for PGP3. Each square within each panel represents the age-group-specific sero-prevalence, the lines above and below each square indicate the 95% binomial confidence intervals associated with the reported age-group specific prevalence. The titles within each panel report the transmission scenario from which the parameter estimates and fits have been obtained. Solid black lines were generated using the median parameter estimates from each model fit. Solid black lines running through the sero-prevalence data were generated with the median parameter estimates from each model fit. The shaded grey regions about the black line represent 95% credible intervals of the model predictions. Uncertainty was generated by drawing 500 independent samples from the posterior distribution and re-running the model with these new values, this was done to understand variation in the model prediction.

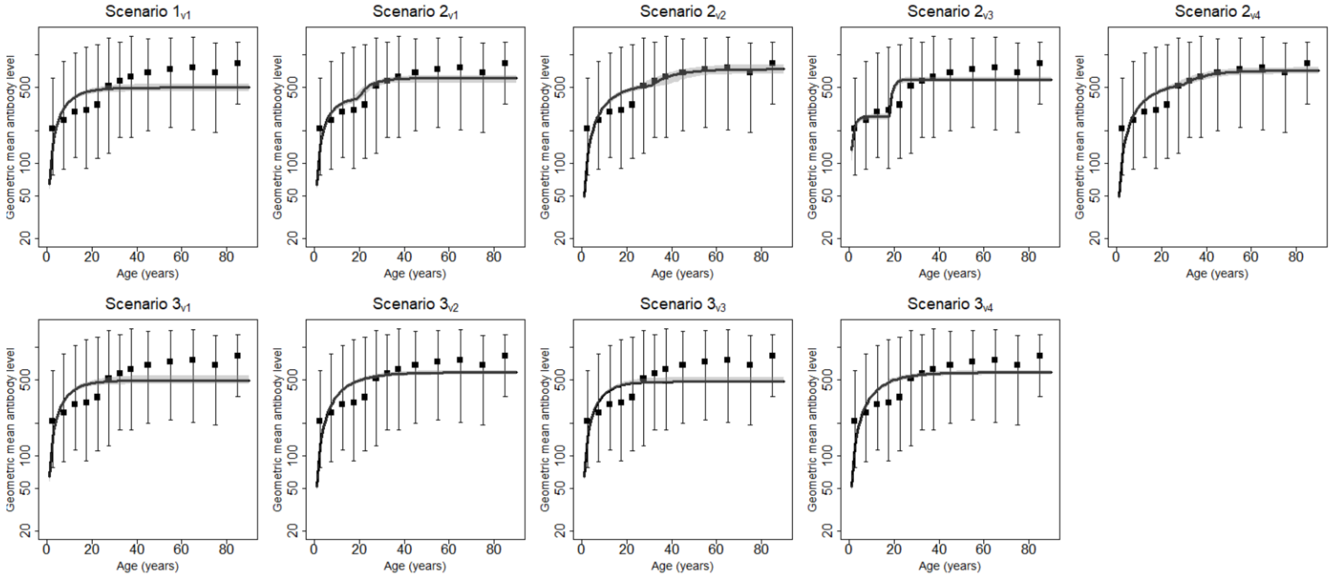

Supplementary Figure 14: Fits of each antibody acquisition model to geometric mean antibody level by age-group from The Gambia, Lower River Region for PGP3. Each square within each panel represents the age-group-specific sero-prevalence, the lines above and below each square indicate the 95% binomial confidence intervals associated with the reported age-group specific prevalence. The titles within each panel report the transmission scenario from which the parameter estimates and fits have been obtained. Solid black

lines were generated using the median parameter estimates from each model fit. Solid black lines running through the sero-prevalence data were generated with the median parameter estimates from each model fit. The shaded grey regions about the black line represent 95% credible intervals of the model predictions. Uncertainty was generated by drawing 500 independent samples from the posterior distribution and re-running the model with these new values, this was done to understand variation in the model prediction.

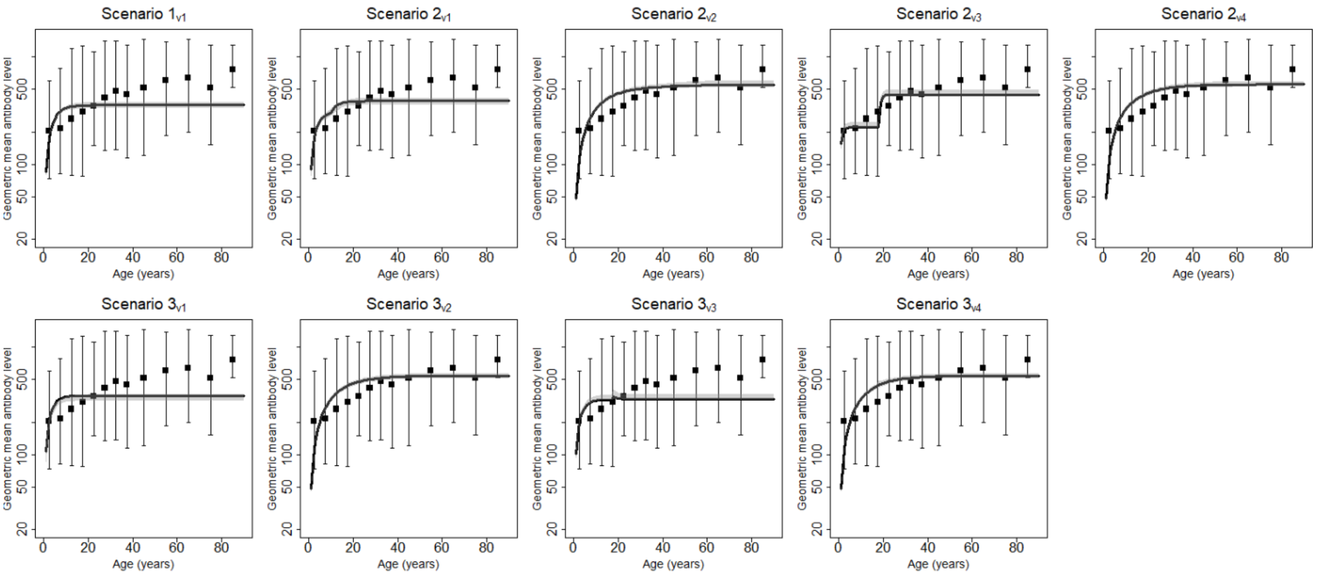

Supplementary Figure 15: Fits of each antibody acquisition model to geometric mean antibody level by age-group from The Gambia, Upper River Region for PGP3. Each square within each panel represents the age-group-specific sero-prevalence, the lines above and below each square indicate the 95% binomial confidence intervals associated with the reported age-group specific prevalence. The titles within each panel report the transmission scenario from which the parameter estimates and fits have been obtained. Solid black lines were generated using the median parameter estimates from each model fit. Solid black lines running through the sero-prevalence data were generated with the median parameter estimates from each model fit. The shaded grey regions about the black line represent 95% credible intervals of the model predictions. Uncertainty was generated by drawing 500 independent samples from the posterior distribution and re-running the model with these new values, this was done to understand variation in the model prediction.

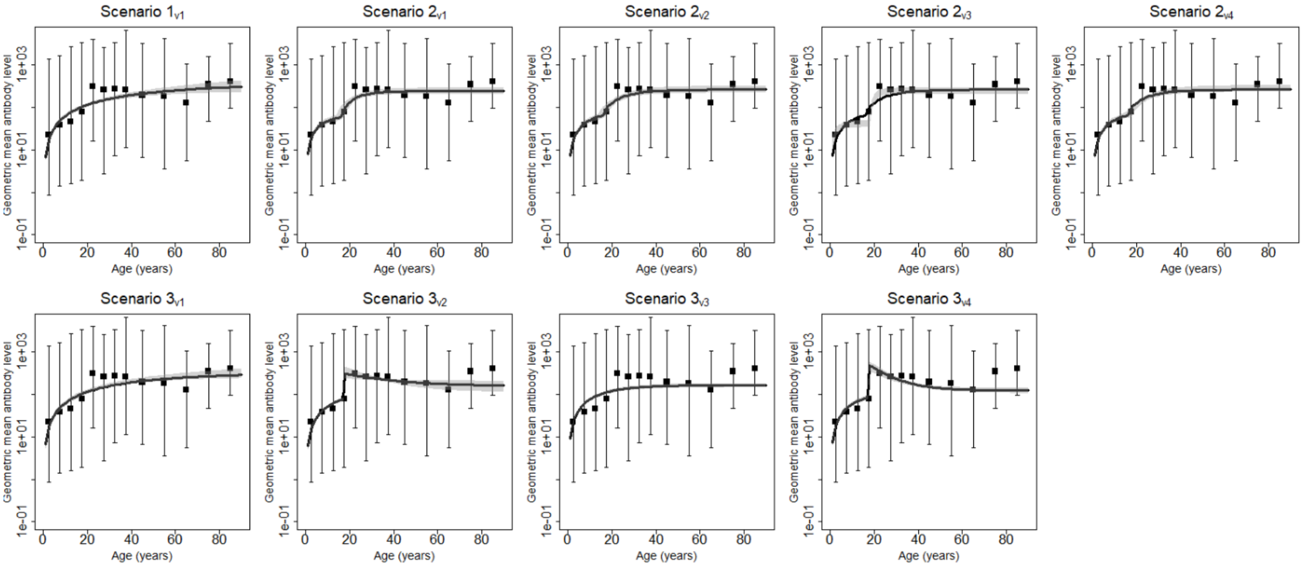

Supplementary Figure 16: Fits of each antibody acquisition model to geometric mean antibody level by age-group from Solomon Islands, Temotu Province for PGP3. Each square within each panel represents the age-group-specific sero-prevalence, the lines above and below each square indicate the 95% binomial

confidence intervals associated with the reported age-group specific prevalence. The titles within each panel report the transmission scenario from which the parameter estimates and fits have been obtained. Solid black lines were generated using the median parameter estimates from each model fit. Solid black lines running through the sero-prevalence data were generated with the median parameter estimates from each model fit. The shaded grey regions about the black line represent 95% credible intervals of the model predictions. Uncertainty was generated by drawing 500 independent samples from the posterior distribution and re-running the model with these new values, this was done to understand variation in the model prediction.

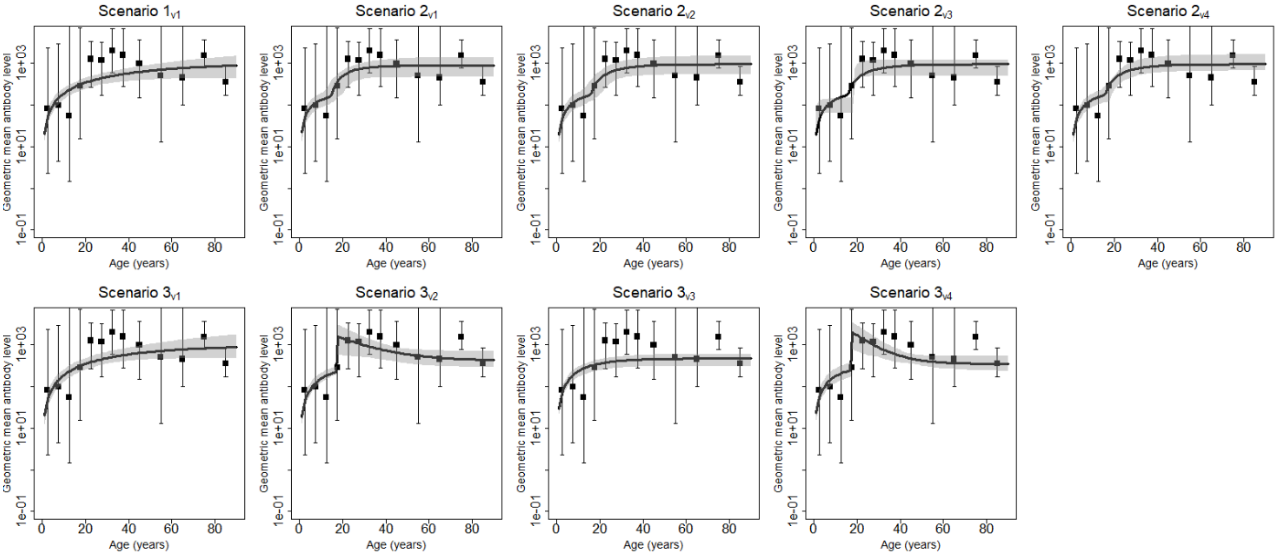

Supplementary Figure 17: Fits of each antibody acquisition model to geometric mean antibody level by age-group from Solomon Islands, Rennell & Bellona Province for PGP3. Each square within each panel represents the age-group-specific sero-prevalence, the lines above and below each square indicate the 95% binomial confidence intervals associated with the reported age-group specific prevalence. The titles within each panel report the transmission scenario from which the parameter estimates and fits have been obtained. Solid black lines were generated using the median parameter estimates from each model fit. Solid black lines running through the sero-prevalence data were generated with the median parameter estimates from each model fit. The shaded grey regions about the black line represent 95% credible intervals of the model predictions. Uncertainty was generated by drawing 500 independent samples from the posterior distribution and re-running the model with these new values, this was done to understand variation in the model prediction.

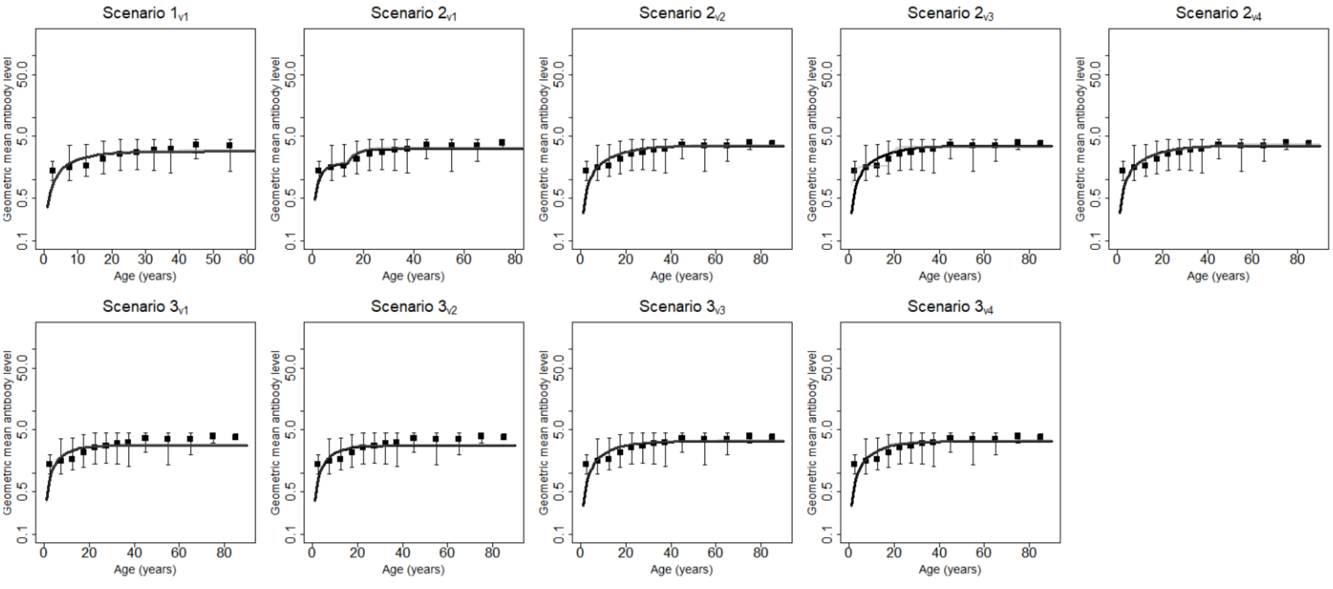

Supplementary Figure 18: Fits of each antibody acquisition model to geometric mean antibody level by age-group from Tanzania, Rombo District for PGP3. Each square within each panel represents the age-group-specific sero-prevalence, the lines above and below each square indicate the 95% binomial confidence intervals associated with the reported age-group specific prevalence. The titles within each panel report the transmission scenario from which the parameter estimates and fits have been obtained. Solid black lines were generated using the median parameter estimates from each model fit. Solid black lines running through the sero-prevalence data were generated with the median parameter estimates from each model fit. The shaded grey regions about the black line represent 95% credible intervals of the model predictions. Uncertainty was generated by drawing 500 independent samples from the posterior distribution and re-running the model with these new values, this was done to understand variation in the model prediction.

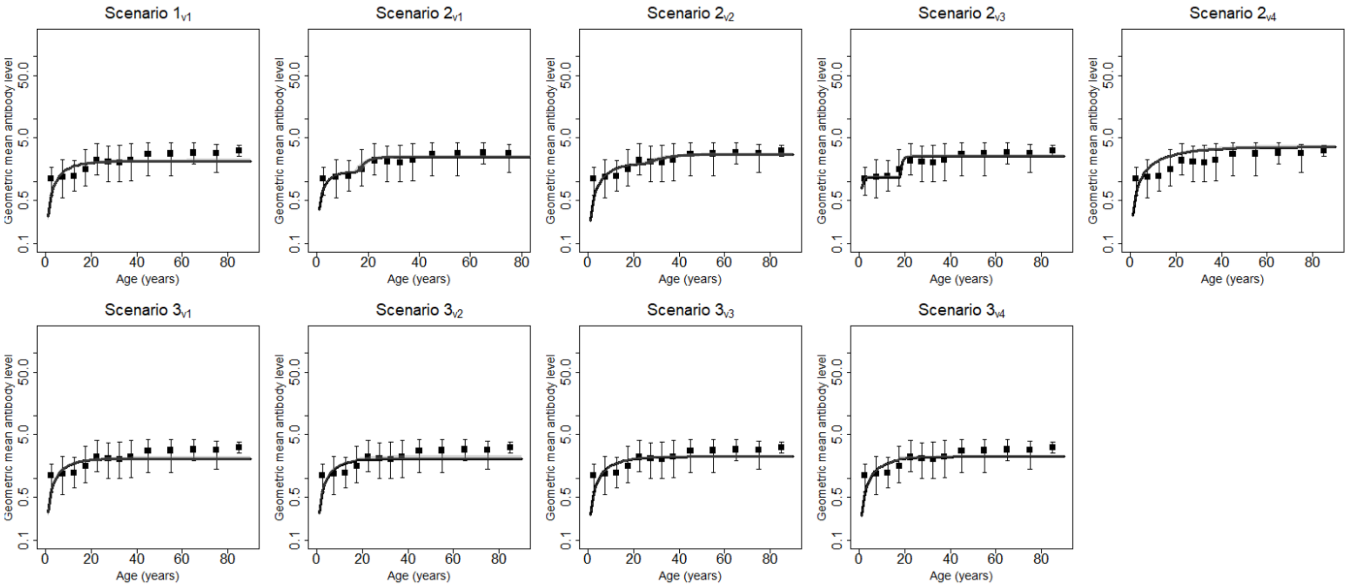

Supplementary Figure 19: Fits of each antibody acquisition model to geometric mean antibody level by age-group from Tanzania, Rombo District for CT694. Each square within each panel represents the age-group-specific sero-prevalence, the lines above and below each square indicate the 95% binomial confidence intervals associated with the reported age-group specific prevalence. The titles within each panel report the transmission scenario from which the parameter estimates and fits have been obtained. Solid black lines were generated using the median parameter estimates from each model fit. Solid black lines running through the sero-prevalence data were generated with the median parameter estimates from each model fit. The shaded grey regions about the black line represent 95% credible intervals of the model predictions. Uncertainty was generated by drawing 500 independent samples from the posterior distribution and re-running the model with these new values, this was done to understand variation in the model prediction.

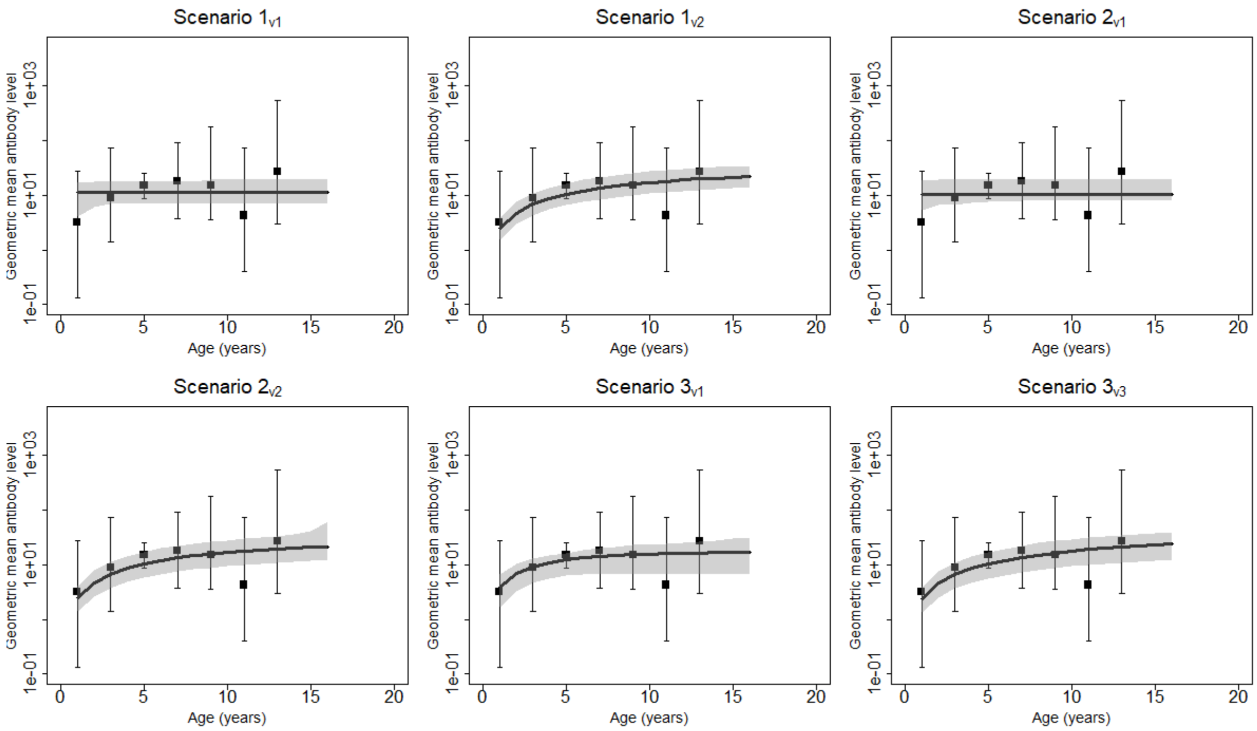

Supplementary Figure 20: Fits of each antibody acquisition model to geometric mean antibody level by age-group from Fiji, Itakeu population for PGP3. Each square within each panel represents the age-group-specific sero-prevalence, the lines above and below each square indicate the 95% binomial confidence intervals associated with the reported age-group specific prevalence. The titles within each panel report the transmission scenario from which the parameter estimates and fits have been obtained. Solid black lines were generated using the median parameter estimates from each model fit. Solid black lines running through the sero-prevalence data were generated with the median parameter estimates from each model fit. The shaded grey regions about the black line represent 95% credible intervals of the model predictions. Uncertainty was generated by drawing 500 independent samples from the posterior distribution and re-running the model with these new values, this was done to understand variation in the model prediction.

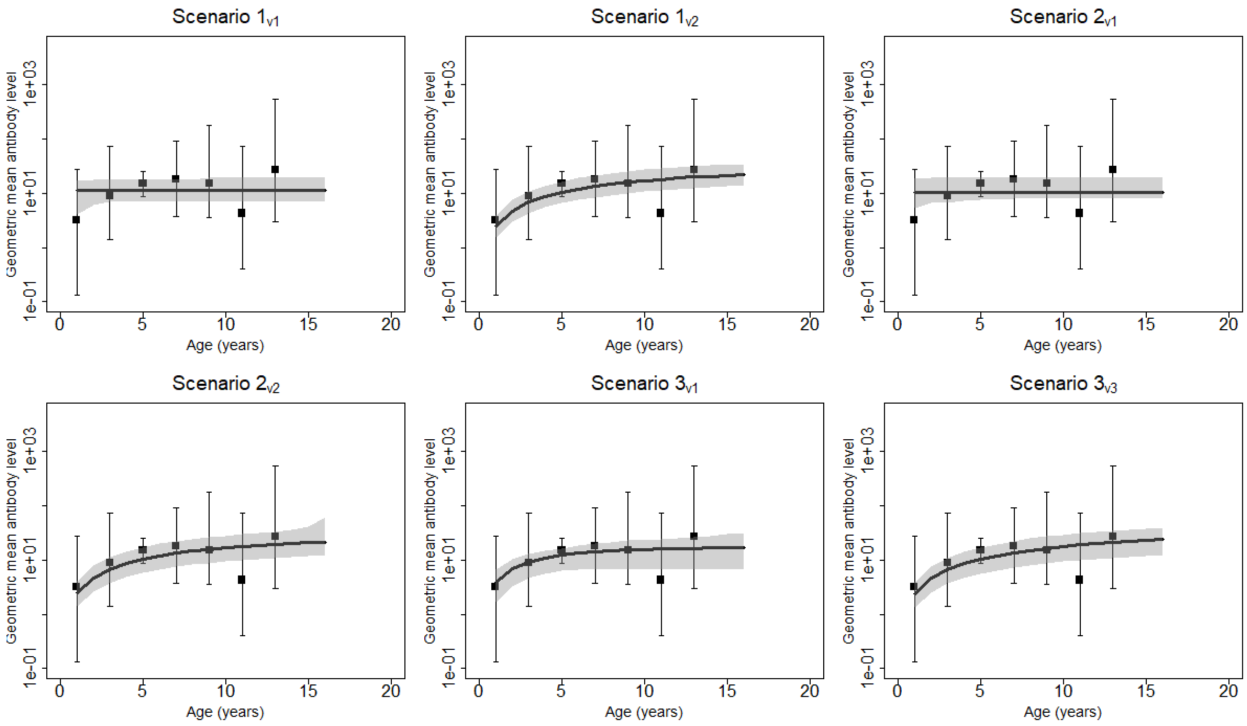

Supplementary Figure 21: Fits of each antibody acquisition model to geometric mean antibody level by age-group from Fiji, Indo-Fijian population for PGP3. Each square within each panel represents the age-group-specific sero-prevalence, the lines above and below each square indicate the 95% binomial confidence intervals associated with the reported age-group specific prevalence. The titles within each panel report the transmission scenario from which the parameter estimates and fits have been obtained. Solid black lines were generated using the median parameter estimates from each model fit. Solid black lines running through the sero-prevalence data were generated with the median parameter estimates from each model fit. The shaded grey regions about the black line represent 95% credible intervals of the model predictions. Uncertainty was generated by drawing 500 independent samples from the posterior distribution and re-running the model with these new values, this was done to understand variation in the model prediction.

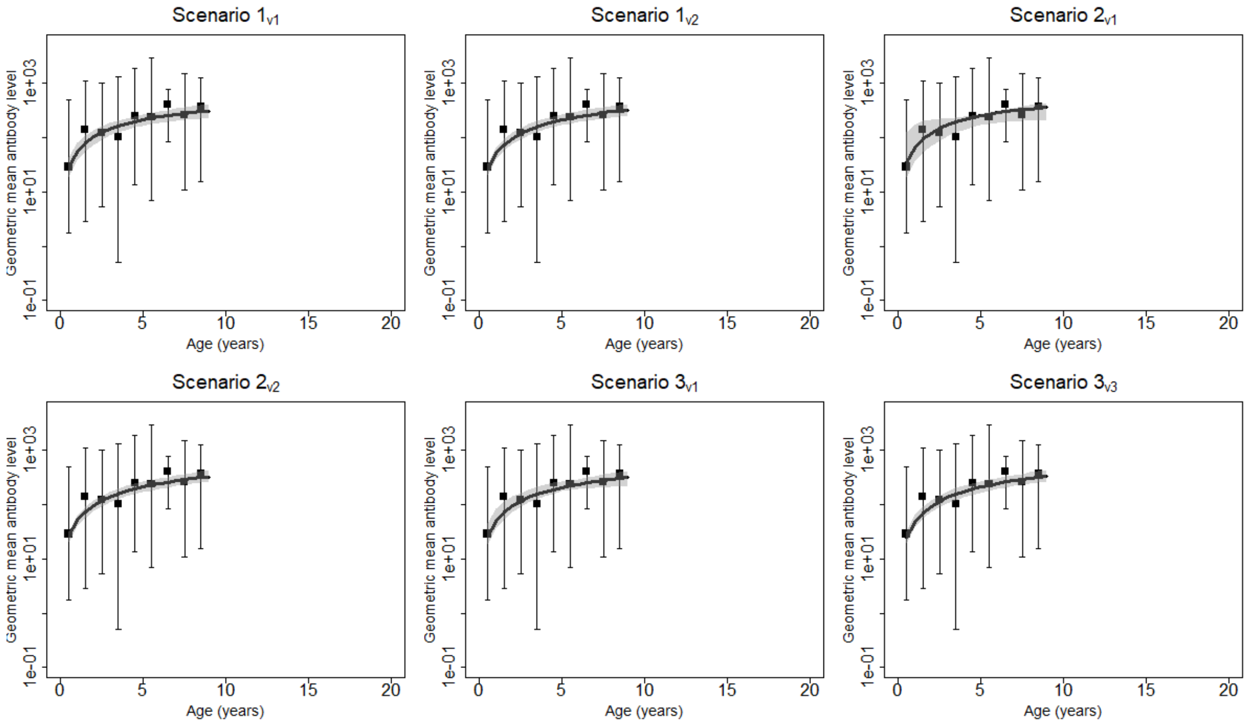

Supplementary Figure 22: Fits of each antibody acquisition model to geometric mean antibody level by age-group from Kiribati for PGP3. Each square within each panel represents the age-group-specific sero-prevalence, the lines above and below each square indicate the 95% binomial confidence intervals associated with the reported age-group specific prevalence. The titles within each panel report the transmission scenario from which the parameter estimates and fits have been obtained. Solid black lines were generated using the median parameter estimates from each model fit. Solid black lines running through the sero-prevalence data were generated with the median parameter estimates from each model fit. The shaded grey regions about the black line represent 95% credible intervals of the model predictions. Uncertainty was generated by drawing 500 independent samples from the posterior distribution and re-running the model with these new values, this was done to understand variation in the model prediction.

Supplementary Tables: Study population characteristics, MCMC chain convergence diagnostics and estimated parameter values for each model for each dataset.

Supplementary Table 1. Characteristics of each study population and the epidemiological prevalence data available. The total number of samples collected, the number that were taken from females, the number of samples that tested sero-positive and the proportion of total samples that that was, and the number of samples that tested TF positive and the proportion of total samples that that was.

Region & age range	Total samples	Total female samples	Proportion sero-positive	Proportion TF positive
Nepal-pre MDA	**659**			
1-9	52		0.59 N=31 (Pgp3)<br>0.55 N=29 (CT694)	0.17 N=9
10-19	144		0.73 N=106<br>0.75 N=109	0.07 N=11
20-29	141		0.80 N=114<br>0.82 N=117	0.03 N=5
30-39	116		0.77 N=90<br>0.79 N=92	0.05 N=6
40 +	206		0.83 N=171<br>0.74 N=154	0.06 N=14
Nepal-post MDA	**640**			
1-9	73		0.02 N=2 (Pgp3)<br>0.05 N=4 (CT694)	0.00 N=0
10-19	218		0.09 N=20<br>0.09 N=20	0.01 N=4
20-29	72		0.34 N=25<br>0.40 N=29	0.00 N=0
30-39	67		0.50 N=34<br>0.73 N=49	0.06 N=4
40 +	210		0.64 N=135<br>0.73 N=154	0.00 N=0
Lower River Region (The Gambia)	**1014**			
1-9	391	199	0.06 N=25	0.03 N=14
10-19	235	131	0.17 N=45	0.01 N=3
20-29	96	71	0.36 N=35	0.01 N=1
30-39	77	55	0.57 N=44	0.01 N=1
40 +	215	117	0.78 N=169	0.00 N=1
Upper River Region (The Gambia)	**825**			
1-9	363	173	0.06 N=22	0.027 N=10
10-19	178	96	0.12 N=22	0.005 N=1
20-29	90	73	0.26 N=24	0.00 N=0
30-39	71	51	0.49 N=35	0.00 N=0
40 +	123	81	0.56 N=70	0.00 N=0
Rombo	**557**			
1-9	172	83	0.03 N=6 (Pgp3)<br>0.01 N=2 (CT694)	0.07 N=13
10-19	188	107	0.10 N=19<br>0.04 N=8	0.00 N=0
20-29	27	19	0.48 N=13<br>0.51 N=14	0.03 N=1
30-39	30	23	0.63 N=19<br>0.40 N=12	0.03 N=1
40 +	140	99	0.82 N=116<br>0.72 N=102	0.00 N=1
Temotu	**1267**			
1-9	386	189	0.14 N=57	0.13 N=52
10-19	290	148	0.23 N=67	0.06 N=20
20-29	133	95	0.65 N=87	0.00 N=0
30-39	180	108	0.31 N=61	0.00 N=1
40 +	278	134	0.92 N=165	0.02 N=6
Rennell & Bellona	**234**			
1-9	78	40	0.35 N=28	0.18 N=14
10-19	46	24	0.06 N=3	0.04 N=2
20-29	21	16	0.75 N=18	0.00 N=0
30-39	20	15	1.00 N=20	0.05 N=1
40 +	69	42	0.88 N= 61	0.00 N=0
Fiji-iTaukei	**477**			
1-9	378	206	0.24 N=91	-
10-15	99	46	0.69 N=69	-
Fiji-Indo-Fijian	**89**			
1-9	54	28	0.01 N=1	-

10-15	35	18	0.05 N=2	-
Kiritbati	**397**			
1-9	397	199	0.48 N=187	0.29 N = 119

Convergence diagnostics for the best fitting model for each site

We calculated the Gelman-Rubin statistic for each parameter for each best fitting model to ensure parameter estimates were corrected and convergence had been achieved (3). We also calculated the Effective Sample Size (ESS) for each chain to ensure effective estimation of the posterior.

**Site**	**λ_T**	**γ**	**ρ**	**t_c**	**λ_{UG}**
Nepal-PGP3	1.00<br>> 500	1.00<br>> 500	1.00<br>> 500	1.01<br>> 500	-
Nepal-CT694	1.00<br>> 500	1.00<br>> 500	1.00<br>> 500	1.03<br>> 500	-
Rombo-PGP3	1.37<br>> 350	1.08<br>> 350	-	-	1.00<br>> 350
Rombo-CT694	1.34<br>> 350	1.02<br>> 350	-	-	1.00<br>> 350
Lower River Region	1.00<br>> 500	1.00<br>> 500	-	-	1.00<br>> 500
Upper River Region	1.00<br>> 500	1.00<br>> 500	-	-	1.00<br>> 500
Solomon Islands-Temotu	1.01<br>> 350	1.00<br>> 350	-	-	1.10<br>> 350
Solomon Islands-RenBel	1.01<br>> 350	1.01<br>> 350	-	-	1.01<br>> 350
Fiji-iTak	1.00<br>> 500	-	-	-	-
Fiji-Indo-Fijian	1.00<br>> 500	-	-	-	-
Kiribati	1.00<br>> 350	1.03<br>> 350	1.01<br>> 350	-	-

Supplementary Table 2: Convergence diagnostics for each of the best fitting models for each site. The number in the first row for each site is the Gelman Rubin statistic for each parameter and the second number is the ESS for each chain for each parameter.

Parameter estimates for each sero-catalytic model for each site

**Scenario**	**λ_T**	**γ**	**ρ**	**t_c**	**DIC**	**pD**	**AIC**
Scenario 1_{v1}	0.044<br>(0.038-0.051)	-	0.012<br>(0.008-0.019)	-	1617.40	1.35	1615.53
Scenario 2_{v1}	0.143<br>(0.107-0.215)	0.053<br>(0.031-0.084)	0.026<br>(0.020-0.032)	16.06<br>(13.57-17.69)	**1325.11**	3.51	**1343.16**

Supplementary Table 3: Parameter estimates for 2 cross-sections from Nepal for PGP3 for the sero-catalytic models evaluated. The first value presented in each cell is the median parameter estimate and the values in the brackets below are the 95% credible intervals for the estimated paramater (the 2.5% and 97.5% percentile of the posterior distribution). DIC of the best performing selected model is highlighted in red.

**Scenario**	**λ_T**	**γ**	**ρ**	**t_c**	**DIC**	**pD**	**AIC**
Scenario 1_{v1}	0.047<br>(0.041-0.053)	-	0.008<br>(0.005-0.013)	-	1531.20	2.00	1527.92
Scenario 2_{v1}	0.142<br>(0.103-0.207)	0.062<br>(0.037-0.097)	0.017<br>(0.013-0.021)	16.84<br>(14.93-18.65)	**1252.95**	3.45	**1271.11**

Supplementary Table 4: Parameter estimates for 2 cross-sections from Nepal for CT694 for the sero-catalytic models evaluated. The first value presented in each cell is the median parameter estimate and the values in the brackets below are the 95% credible intervals for the estimated parameters (the 2.5% and 97.5% percentile of the posterior distribution). DIC of the best performing selected model is highlighted in red.

Scenario	λ_T	γ	ρ	t_c	λ_{UG}	DIC	pD	AIC
Scenario 1_{v1}	0.021 (0.019-0.024)	-	0.0006 (0.00001-0.002)	-	-	885.11	1.00	895.33
Scenario 2_{v1}	0.097 (0.034-0.324)	0.158 (0.049-0.347)	0.008 (0.002-0.015)	23.28 (9.73-27.98)	-	852.30	3.86	866.15
Scenario 2_{v2}	0.364 (0.157-0.873)	0.051 (0.021-0.893)	-	15.73 (13.06-23.32)	-	904.37	2.37	903.35
Scenario 2_{v3}	0.029 (0.013-0.244)	0.478 (0.061-0.977)	0.040 (0.007-0.078)	25.11 (0.96-48.89)	0.014 (0.004-0.033)	862.03	2.62	887.89
Scenario 2_{v4}	0.101 (0.021-0.370)	0.152 (0.040-0.721)	-	35.81 (22.38-58.16)	0.060 (0.039-0.079)	864.56	2.29	884.75
Scenario 3_{v1}	0.038 (0.028-0.052)	0.248 (0.097-0.437)	0.006 (0.001-0.010)	-	-	866.98	2.60	876.41
Scenario 3_{v2}	0.025 (0.018-0.040)	0.408 (0.180-0.626)	0.012 (0.004-0.035)	-	0.031 (0.004-0.073)	864.23	2.45	879.36
Scenario 3_{v3}	0.086 (0.067-0.106)	0.051 (0.007-0.161)	-	-	-	902.13	1.32	907.69
Scenario 3_{v4}	0.021 (0.013-0.036)	0.677 (0.268-0.984)	-	-	0.067 (0.049-0.090)	866.64	3.20	878.45

Supplementary Table 5: Parameter estimates for 1 cross-section from The Gambia, LRR for PGP3 for the sero-catalytic models evaluated. The first value presented in each cell is the median parameter estimate and the values in the brackets below are the 95% credible intervals for the estimated parameters (the 2.5% and 97.5% percentile of the posterior distribution). DIC of the best performing selected model is highlighted in red.

Scenario	λ_T	γ	ρ	t_c	λ_{UG}	DIC	pD	AIC
Scenario 1_{v1}	0.015 (0.013-0.018)	-	0.002 (0.0001-0.012)	-	-	682.66	0.98	692.60
Scenario 2_{v1}	0.048 (0.018-0.173)	0.015 (0.002-0.031)	0.013 (0.008-0.018)	19.32 (8.64-45.48)	-	662.84	4.86	683.25
Scenario 2_{v2}	0.103 (0.048-1.209)	0.128 (0.012-0.303)	-	20.09 (12.83-29.11)	-	620.83	2.11	680.59
Scenario 2_{v3}	0.057 (0.015-0.374)	0.278 (0.035-0.875)	0.050 (0.011-0.177)	32.50 (2.87-57.11)	0.052 (0.014-0.212)	679.73	2.32	704.74
Scenario 2_{v4}	0.039 (0.012-0.206)	0.334 (0.061-0.974)	-	31.96 (5.01-58.05)	0.026 (0.006-0.041)	676.47	1.63	698.11
Scenario 3_{v1}	0.029 (0.020-0.051)	0.316 (0.119-0.503)	0.005 (0.014-0.027)	-	-	681.03	2.35	691.58
Scenario 3_{v2}	0.030 (0.014-0.119)	0.551 (0.104-0.941)	0.011 (0.022-0.359)	-	0.096 (0.027-0.305)	674.23	1.47	696.27
Scenario 3_{v3}	0.044 (0.031-0.061)	0.204 (0.058-0.407)	-	-	-	684.61	1.38	690.30
Scenario 3_{v4}	0.023 (0.010-0.184)	0.591 (0.112-0.893)	-	-	0.063 (0.015-0.595)	678.64	1.94	688.60

Supplementary Table 6: Parameter estimates for 1 cross-section from The Gambia, URR for PGP3 for the sero-catalytic models evaluated. The first value presented in each cell is the median parameter estimate and the values in the brackets below are the 95% credible intervals for the estimated parameters (the 2.5% and 97.5% percentile of the posterior distribution). DIC of the best performing selected model is highlighted in red.

Scenario	λ_T	γ	ρ	t_c	λ_{UG}	DIC	pD	AIC
Scenario 1_{v1}	0.021 (0.017-0.024)	-	0.0006 (0.00005-0.002)	-	-	427.60	0.96	437.60
Scenario 2_{v1}	0.056 (0.035-0.124)	0.113 (0.051-0.222)	0.004 (0.0003-0.011)	13.72 (10.03-19.06)	-	378.39	2.99	384.23
Scenario 2_{v2}	0.087 (0.046-0.193)	0.093 (0.038-0.205)	-	17.94 (13.44-19.73)	-	394.67	1.53	405.12
Scenario 2_{v3}	0.019 (0.009-0.446)	0.050 (0.019-0.809)	0.025 (0.005-0.115)	40.96 (2.87-59.37)	0.096 (0.029-0.341)	382.05	2.09	405.96
Scenario 2_{v4}	0.032 (0.008-0.211)	0.230 (0.039-0.965)	-	36.23 (3.36-59.13)	0.097 (0.070-0.135)	380.43	1.04	401.28
Scenario 3_{v1}	0.040 (0.030-0.054)	0.036 (0.002-0.183)	0.003 (0.0007-0.009)	-	-	382.28	2.50	392.14
Scenario 3_{v2}	0.021 (0.010-0.041)	0.160 (0.005-0.706)	0.017 (0.005-0.046)	-	0.067 (0.032-0.159)	379.18	1.48	395.97
Scenario 3_{v3}	0.084 (0.067-0.105)	0.013 (0.0006-0.064)	-	-	-	426.86	0.80	432.29
Scenario 3_{v4}	0.022 (0.009-0.041)	0.177 (0.019-0.881)	-	-	0.092 (0.061-0.127)	379.74	1.74	391.69

Supplementary Table 7: Parameter estimates for 1 cross-section from Tanzania, Rombo District for PGP3 for the sero-catalytic models evaluated. The first value presented in each cell is the median parameter estimate and the values in the brackets below are the 95% credible intervals for the estimated parameters (the 2.5% and 97.5% percentile of the posterior distribution). DIC of the best performing selected model is highlighted in red.

Scenario	λ_T	γ	ρ	t_c	λ_{UG}	DIC	pD	AIC
Scenario 1_{v1}	0.014 (0.013-0.017)	-	0.0005 (0.000009-0.003)	-	-	386.87	0.67	397.26
Scenario 2_{v1}	0.038 (0.026-0.071)	0.040 (0.008-0.106)	0.004 (0.0002-0.013)	12.94 (11.08-15.18)	-	322.37	2.99	326.13
Scenario 2_{v2}	0.085 (0.058-0.138)	0.022 (0.006-0.056)	-	14.07 (12.20-16.88)	-	329.43	2.33	327.76
Scenario 2_{v3}	0.119 (0.07-0.529)	0.135 (0.024-0.934)	0.808 (0.034-1.417)	34.71 (2.18-59.20)	1.423 (0.080-2.283)	346.30	0.89	358.07
Scenario 2_{v4}	0.017 (0.003-0.095)	0.136 (0.021-0.585)	-	29.74 (3.49-58.64)	0.048 (0.024-0.066)	326.69	0.58	345.70
Scenario 3_{v1}	0.022 (0.018-0.031)	0.048 (0.004-0.145)	0.002 (0.0001-0.008)	-	-	382.28	2.50	392.14
Scenario 3_{v2}	0.009 (0.004-0.016)	0.117 (0.060-0.152)	0.013 (0.001-0.028)	-	0.038 (0.018-0.060)	328.04	1.76	345.15
Scenario 3_{v3}	0.041 (0.033-0.050)	0.011 (0.0003-0.058)	-	-	-	352.80	0.82	358.01
Scenario 3_{v4}	0.008 (0.004-0.016)	0.172 (0.002-0.567)	-	-	0.048 (0.031-0.062)	326.74	1.00	339.32

Supplementary Table 8: Parameter estimates for 1 cross-section from Tanzania, Rombo District for CT694 for the sero-catalytic models evaluated. The first value presented in each cell is the median parameter estimate and the values in the brackets below are the 95% credible intervals for the estimated parameters (the 2.5% and 97.5% percentile of the posterior distribution). DIC of the best performing selected model is highlighted in red.

Scenario	λ_T	γ	ρ	t_c	λ_{UG}	DIC	pD	AIC
Scenario 1_{v1}	0.035 (0.029-0.041)	-	0.016 (0.009-0.024)	-	-	1451.86	1.32	1461.74
Scenario 2_{v1}	1.19 (0.319-1.649)	0.024 (0.016-0.090)	0.033 (0.027-0.039)	18.12 (16.71-20.89)	-	1410.08	2.61	1419.16
Scenario 2_{v2}	0.830 (0.183-1.762)	0.032 (0.014-0.150)	-	19.74 (16.55-21.89)	-	1416.50	0.36	1417.45
Scenario 2_{v3}	0.119 (0.07-0.529)	0.408 (0.107-0.955)	0.202 (0.102-0.505)	30.99 (4.23-58.55)	0.279 (0.135-0.701)	1410.71	1.52	1437.59
Scenario 2_{v4}	0.322 (0.111-0.393)	0.082 (0.060-0.254)	-	17.94 (15.76-21.06)	0.003 (0.00006-0.014)	1420.20	2.31	1439.34
Scenario 3_{v1}	0.081 (0.055-0.119)	0.283 (0.136-0.514)	0.027 (0.020-0.036)	-	-	1444.00	2.56	1454.46
Scenario 3_{v2}	0.243 (0.083-0.495)	0.376 (0.087-0.906)	0.418 (0.195-0.771)	-	0.612 (0.261-1.201)	1405.39	1.88	1423.75
Scenario 3_{v3}	0.077 (0.056-0.104)	0.293 (0.153-0.501)	-	-	-	1442.22	1.27	1447.92
Scenario 3_{v4}	0.045 (0.028-0.075)	0.585 (0.218-0.986)	-	-	0.021 (0.001-0.0641)	1440.54	1.83	1452.35

Supplementary Table 9: Parameter estimates for 1 cross-section from Solomon Islands, Temotu district for PGP3 for the sero-catalytic models evaluated. The first value presented in each cell is the median parameter estimate and the values in the brackets below are the 95% credible intervals for the estimated parameters (the 2.5% and 97.5% percentile of the posterior distribution). DIC of the best performing selected model is highlighted in red.

Scenario	λ_T	γ	ρ	t_c	λ_{UG}	DIC	pD	AIC
Scenario 1_{v1}	0.076 (0.058-0.100)	-	0.009 (0.003-0.021)	-	-	253.93	1.28	263.90
Scenario 2_{v1}	0.315 (0.071-1.103)	0.019 (0.054-0.905)	0.010 (0.004-0.022)	16.63 (0.23-33.64)	-	**233.13**	3.94	**253.27**
Scenario 2_{v2}	0.480 (0.089-2.061)	0.170 (0.043-0.936)	-	17.75 (0.26-42.46)	-	262.14	4.23	256.02
Scenario 2_{v3}	0.948 (0.308-2.42)	0.471 (0.128-0.962)	0.724 (0.374-0.505)	29.08 (0.95-58.69)	5.684 (3.240-7.301)	236.42	1.09	264.43
Scenario 2_{v4}	1.080 (0.345-1.737)	0.063 (0.034-0.204)	-	40.47 (17.13-58.65)	0.195 (0.083-0.389)	245.27	1.18	267.46
Scenario 3_{v1}	0.109 (0.055-0.119)	0.610 (0.23-0.95)	0.010 (0.004-0.020)	-	-	253.71	1.45	265.54
Scenario 3_{v2}	1.191 (0.381-2.247)	0.462 (0.188-0.981)	0.985 (0.353-2.176)	-	7.344 (2.828-9.849)	238.89	1.05	256.69
Scenario 3_{v3}	0.150 (0.092-0.286)	0.529 (0.195-0.972)	-	-	-	260.79	1.47	266.79
Scenario 3_{v4}	0.092 (0.061-0.170)	0.746 (0.319-0.995)	-	-	0.255 (0.085-0.499)	247.34	1.00	258.41

Supplementary Table 10: Parameter estimates for 1 cross-section from Solomon Islands, Rennell & Bellona Province district for PGP3 for the sero-catalytic models evaluated. The first value presented in each cell is the median parameter estimate and the values in the brackets below are the 95% credible intervals for the estimated parameters (the 2.5% and 97.5% percentile of the posterior distribution). DIC of the best performing selected model is highlighted in red.

Scenario	λ_T	γ	ρ	t_c	λ_{UG}	DIC	pD	AIC
Scenario 1_{v1}	0.208 (0.163-0.293)	-	0.044 (0.002-0.131)	-	-	454.07	1.04	464.22
Scenario 1_{v2}	0.194 (0.167-0.225)	-	-	-	-	471.73	0.41	477.49
Scenario 2_{v1}	0.413 (0.206-1.555)	0.302 (0.057-0.928)	0.088 (0.010-0.186)	1.78 (0.48-9.62)	-	471.11	3.40	475.44
Scenario 2_{v2}	0.246 (0.183-1.80)	0.475 (0.070-0.969)	-	5.14 (0.13-9.83)	-	453.18	-24.90	471.43
Scenario 3_{v1}	1.460 (0.153-2.679)	0.045 (0.021-0.438)	0.013 (0.006-0.025)	-	-	450.53	1.66	461.76
Scenario 3_{v3}	1.080 (0.345-1.737)	0.063 (0.034-0.204)	-	-	-	**453.45**	0.89	**459.55**

Supplementary Table 11: Parameter estimates for 1 cross-section from Kiribati for PGP3 for the sero-catalytic models evaluated. The first value presented in each cell is the median parameter estimate and the values in the brackets below are the 95% credible intervals for the estimated parameters (the 2.5% and 97.5% percentile of the posterior distribution). DIC of the best performing selected model is highlighted in red.

Scenario	λ_T	γ	ρ	t_c	λ_{UG}	DIC	pD	AIC
Scenario 1_{v1}	2.316 (0.255-3.572)	-	6.729 (0.639-9.879)	-	-	541.40	0.36	551.13
Scenario 1_{v2}	0.053 (0.044-0.063)	-	-	-	-	554.99	0.41	560.75
Scenario 2_{v1}	1.428 (0.211-4.170)	0.410 (0.098-0.956)	1.483 (0.317-3.680)	7.692 (0.64-14.62)	-	533.41	1.07	547.95
Scenario 2_{v2}	0.309 (0.055-1.226)	0.171 (0.042-0.941)	-	13.75 (5.50-14.93)	-	418.05	0.84	557.90
Scenario 3_{v1}	3.264 (0.313-8.273)	0.474 (0.315-0.980)	4.923 (0.504-9.279)	-	-	540.02	0.06	553.16
Scenario 3_{v3}	0.058 (0.046-0.080)	0.045 (0.021-0.438)	-	-	-	557.37	1.27	562.77

Supplementary Table 12: Parameter estimates for 1 cross-section from the iTakeu population in Fiji for PGP3 for the sero-catalytic models evaluated. The first value presented in each cell is the median parameter estimate and the values in the brackets below are the 95% credible intervals for the estimated parameters (the 2.5% and 97.5% percentile of the posterior distribution). DIC of the best performing selected model is highlighted in red.

Scenario	λ_T	γ	ρ	t_c	λ_{UG}	DIC	pD	AIC
Scenario 1_{v1}	2.261 (0.057-0.870)	-	6.993 (1.667-9.879)	-	-	27.38	0.35	37.87
Scenario 1_{v2}	0.006 (0.001-0.014)	-	-	-	-	23.30	0.35	31.29
Scenario 2_{v1}	0.770 (0.088-5.198)	0.179 (0.098-0.956)	4.200 (1.499-5.798)	7.77 (0.54-14.65)	-	23.41	0.42	33.75
Scenario 2_{v2}	0.019 (0.003-0.356)	0.184 (0.010-0.685)	-	10.91 (1.85-14.82)	-	19.88	0.21	**27.76**
Scenario 3_{v1}	1.198 (0.142-4.221)	0.474 (0.315-0.980)	6.718 (1.133-9.916)	-	-	24.79	0.35	39.63

Scenario 3_{v3}	0.010 (0.002-0.032)	0.392 (0.003-0.988)	-	-	-	25.54	0.39	33.02

Supplementary Table 13: Parameter estimates for 1 cross-section from the Indo-Fijian population in Fiji for PGP3 for the sero-catalytic models evaluated. The first value presented in each cell is the median parameter estimate and the values in the brackets below are the 95% credible intervals for the estimated parameters (the 2.5% and 97.5% percentile of the posterior distribution). DIC of the best performing selected model is highlighted in red.

Parameter estimates for each antibody acquisition model for each site

Scenario	λ_T	γ	ρ	t_c	σ	DIC	pD
Scenario 1_{v1}	0.036 (0.032-0.051)	-	0.012 (0.008-0.019)	-	0.597 (0.465-0.791)	1617.40	1.35
Scenario 2_{v1}	0.362 (0.327-0.403)	0.471 (0.433 -0.515)	0.093 (0.083-0.106)	13.39 (11.09-15.16)	0.514 (0.493-0.537)	1325.11	3.51

Supplementary Table 14: Parameter estimates for 2 cross-sections from Nepal for PGP3 for the antibody acquisition models evaluated. The first value presented in each cell is the median parameter estimate and the values in the brackets below are the 95% credible intervals for the estimated parameters (the 2.5% and 97.5% percentile of the posterior distribution). DIC of the best performing selected model is highlighted in red.

Scenario	λ_T	γ	ρ	t_c	σ	DIC	pD
Scenario 1_{v1}	0.047 (0.041-0.053)	-	0.008 (0.005-0.013)	-	0.603 (0.415-0.517)	1531.20	2.00
Scenario 2_{v1}	0.373 (0.329-0.437)	0.609 (0.553-0.660)	0.125 (0.109-0.146)	15.50 (11.67-16.54)	0.503 (0.485-0.523)	1252.95	3.45

Supplementary Table 15: Parameter estimates for 2 cross-sections from Nepal for CT694 for the antibody acquisition models evaluated. The first value presented in each cell is the median parameter estimate and the values in the brackets below are the 95% credible intervals for the estimated parameters (the 2.5% and 97.5% percentile of the posterior distribution). DIC of the best performing selected model is highlighted in red.

Scenario	α_T	γ	r	t_c	α_{UG}	σ	DIC	pD
Scenario 1_{v1}	67.844 (60.108-78.077)	-	0.136 (0.112-0.167)	-	-	0.685 (0.657-0.716)	13706.67	2.30
Scenario 2_{v1}	962.922 (825.755-998.210)	0.071 (0.064-0.084)	0.168 (0.154-0.198)	19.44 (16.37-21.80)	-	0.647 (0.629-0.696)	13606.40	-4.15
Scenario 2_{v2}	374.859 (172.941-860.259)	0.137 (0.060-0.299)	-	31.14 (23.78-41.24)	-	0.677 (0.650-0.706)	13695.91	2.89
Scenario 2_{v3}	311.258 (176.178-590.285)	0.583 (0.266-0.967)	0.675 (0.471-0.908)	34.49 (3.75-54.12)	215.726 (146.201-296.743)	0.167 (0.591-0.645)	13504.80	2.34
Scenario 2_{v4}	273.536 (130.612-349.726)	0.186 (0.145-0.393)	-	29.71 (24.73-37.64)	2.198 (0.109-8.751)	0.679 (0.649-0.711)	13698.10	2.98
Scenario 3_{v1}	93.668 (65.065-167.914)	0.668 (0.257-0.976)	0.144 (0.117-0.177)	-	-	0.683 (0.655-0.715)	13708.11	2.89
Scenario 3_{v2}	179.516 (134.004-204.238)	0.921 (0.741-0.997)	0.596 (0.465-0.737)	-	187.174 (146.995-249.043)	0.617 (0.591-0.645)	13710.14	2.48
Scenario 3_{v3}	54.451 (52.129-56.778)	0.974 (0.871-0.999)	-	-	-	0.688 (0.659-0.719)	13721.58	1.94
Scenario 3_{v4}	54.430 (50.320-70.913)	0.867 (0.449-0.995)	-	-	9.891 (4.351-16.149)	0.684 (0.655-0.715)	13724.60	2.83

Supplementary Table 16: Parameter estimates for 1 cross-sections from The Gambia, LRR for PGP3 for the antibody acquisition models evaluated. The first value presented in each cell is the median parameter estimate and the values in the brackets below are the 95% credible intervals for the estimated parameters (the

2.5% and 97.5% percentile of the posterior distribution). DIC of the best performing selected model is highlighted in red.

Scenario	α_T	γ	r	t_c	α_{UG}	σ	DIC	pD
Scenario 1_{v1}	97.309 (88.213-99.869)	-	0.274 (0.239-0.295)	-	-	0.697 (0.665-0.732)	10853.66	1.81
Scenario 2_{v1}	930.60 (654.08-997.47)	0.116 (0.095-0.159)	0.350 (0.288-0.419)	9.75 (7.78-12.08)	-	0.677 (0.649-0.707)	10815.73	3.01
Scenario 2_{v2}	105.827 (51.472-331.289)	0.475 (0.151-0.979)	-	43.20 (21.26-59.27)	-	0.748 (0.714-0.784)	10969.46	1.47
Scenario 2_{v3}	443.998 (262.849-962.978)	0.598 (0.370-0.971)	1.205 (0.925-1.721)	49.89 (7.08-59.48)	26.045 (1.678-58.719)	0.635 (0.606-0.668)	10703.96	-0.62
Scenario 2_{v4}	53.261 (49.065-62.396)	0.943 (0.685-0.992)	-	18.46 (10.27-28.35)	1.056 (0.039-4.784)	0.749 (0.714-0.790)	10973.14	2.35
Scenario 3_{v1}	184.835 (108.715-373.339)	0.621 (0.172-0.985)	0.370 (0.280-0.557)	-	-	0.696 (0.662-0.730)	10484.29	-1.17
Scenario 3_{v2}	237.556 (154.649-277.305)	0.858 (0.685-0.992)	0.821 (0.562-1.076)	-	173.867 (105.405-234.512)	0.637 (0.607-0.669)	10852.40	1.69
Scenario 3_{v3}	50.510 (47.956-53.237)	0.985 (0.932-0.999)	-	-	-	0.748 (0.715-0.788)	10974.08	2.37
Scenario 3_{v4}	56.808 (49.249-95.463)	0.769 (0.060-0.991)	-	-	-	0.750 (0.715-0.789)	10972.11	1.98

Supplementary Table 17: Parameter estimates for 1 cross-sections from The Gambia, URR for PGP3 for the antibody acquisition models evaluated. The first value presented in each cell is the median parameter estimate and the values in the brackets below are the 95% credible intervals for the estimated parameters (the 2.5% and 97.5% percentile of the posterior distribution). DIC of the best performing selected model is highlighted in red.

Scenario	α_T	γ	r	t_c	α_{UG}	σ	DIC	pD
Scenario 1_{v1}	6.792 (5.843-7.977)	-	0.017 (0.005-0.030)	-	-	1.809 (1.731-1.890)	14531.9	2.42
Scenario 2_{v1}	202.556 (157.658-257.660)	0.042 (0.032-0.056)	0.131 (0.116-0.151)	16.14 (13.76-17.59)	-	1.777 (1.709-1.846)	14491.9	3.31
Scenario 2_{v2}	77.688 (52.072-107.361)	0.098 (0.067-0.150)	-	15.93 (12.90-17.69)	-	1.783 (1.712-1.858)	14499.03	3.12
Scenario 2_{v3}	51.633 (17.897-119.624)	0.351 (0.147-0.947)	0.517 (0.283-0.782)	13.51 (1.78-58.54)	103.652 (54.086–156.295)	1.767 (1.697-1.846)	14478.0	-0.68
Scenario 2_{v4}	16.790 (7.715-73.366)	0.465 (0.104-0.968)	-	19.41 (6.93-58.82)	15.866 (1.805-21.357)	1.784 (1.711-1.862)	14501.5	1.49
Scenario 3_{v1}	11.691 (6.670-31.894)	0.486 (0.172-0.985)	0.028 (0.013-0.053)	-	-	1.807 (1.732-1.881)	14530.9	1.95
Scenario 3_{v2}	45.797 (21.834-99.934)	0.280 (0.022-0.951)	0.399 (0.234-0.841)	-	77.883 (42.348-169.332)	1.767 (1.695-1.844)	14496.10	3.32
Scenario 3_{v3}	12.170 (10.960-13.596)	0.054 (0.002-0.289)	-	-	-	1.829 (1.754-1.907)	14557.0	2.02
Scenario 3_{v4}	9.894 (7.499-15.372)	0.594 (0.041-0.963)	-	-	18.134 (13.768-12.118)	1.785 (1.713-1.861)	14518.10	2.84

Supplementary Table 18: Parameter estimates for 1 cross-sections from Solomon Islands, Temotu Province for PGP3 for the antibody acquisition models evaluated. The first value presented in each cell is the median parameter estimate and the values in the brackets below are the 95% credible intervals for the

estimated parameters (the 2.5% and 97.5% percentile of the posterior distribution). DIC of the best performing selected model is highlighted in red.

Scenario	α_T	γ	r	t_c	α_{UG}	σ	DIC	pD
Scenario 1_{v1}	20.715 (13.870-32.891)	-	0.018 (0.001-0.062)	-	-	2.156 (1.936-2.414)	2924.41	2.57
Scenario 2_{v1}	544.192 (62.288-975.771)	0.042 (0.020-0.316)	0.122 (0.042-0.183)	14.83 (11.63-20.18)	-	2.111 (1.911-2.346)	2915.47	3.93
Scenario 2_{v2}	298.818 (120.380-650.512)	0.069 (0.029-0.200)	-	15.755 (11.579-22.785)	-	2.109 (1.912-2.342)	2916.14	3.40
Scenario 2_{v3}	258.451 (55.864-590.893)	0.383 (0.122-0.938)	1.021 (0.323-1.933)	23.72 (0.08-57.73)	730.184 (225.325-991.689)	2.088 (1.892-2.314)	2910.79	2.51
Scenario 2_{v4}	67.732 (19.488-397.914)	0.307 (0.051-0.935)	-	27.08 (6.14-58.72)	65.757 (8.812-122.785)	2.114 (1.916-2.347)	2915.24	2.13
Scenario 3_{v1}	46.772 (18.952-104.476)	0.298 (0.016-0.937)	0.032 (0.004-0.079)	-	-	2.164 (1.965-2.397)	2923.91	2.15
Scenario 3_{v2}	185.788 (47.533–285.566)	0.310 (0.058-0.922)	0.583 (0.289-1.076)	-	393.424 (213.043-616.153)	2.081 (1.885-2.317)	2919.40	3.15
Scenario 3_{v3}	36.528 (26.537-50.175)	0.252 (0.010-0.908)	-	-	-	2.195 (1.996-2.431)	2928.76	1.91
Scenario 3_{v4}	28.849 (16.613-50.195)	0.480 (0.024-0.969)	-	-	77.970 (36.244-135.504)	2.118 (1.919-2.350)	2922.08	2.12

Supplementary Table 19: Parameter estimates for 1 cross-sections from Solomon Islands, Rennell & Bellona Province for PGP3 for the antibody acquisition models evaluated. The first value presented in each cell is the median parameter estimate and the values in the brackets below are the 95% credible intervals for the estimated parameters (the 2.5% and 97.5% percentile of the posterior distribution). DIC of the best performing selected model is highlighted in red.

Scenario	α_T	γ	ρ	t_c	α_{UG}	σ	DIC	pD
Scenario 1_{v1}	0.377 (0.337-0.423)	-	0.133 (0.111-0.156)	-	-	0.429 (0.404-0.456)	1444.14	2.20
Scenario 2_{v1}	22.430 (12.293-30.434)	0.023 (0.017-0.040)	0.288 (0.251-0.309)	14.17 (13.29-14.93)	-	0.362 (0.347-0.380)	1250.95	1.13
Scenario 2_{v2}	0.470 (0.364-0.768)	0.624 (0.380-0.831)	-	17.28 (13.23-25.56)	-	0.431 (0.406-0.460)	1455.86	2.14
Scenario 2_{v3}	1.650 (1.205-2.381)	0.585 (0.435-0.960)	0.724 (0.604-0.860)	33.05 (6.91-49.52)	1.240 (0.995-1.518)	0.332 (0.314-0.353)	1154.46	-3.92
Scenario 2_{v4}	0.780 (0.314-1.360)	0.376 (0.213-0.941)	-	37.34 (28.12-56.72)	0.021 (0.001-0.053)	0.433 (0.409-0.459)	1454.02	2.33
Scenario 3_{v1}	0.542 (0.378-0.929)	0.642 (0.257-0.985)	0.143 (0.120-0.170)	-	-	0.430 (0.406-0.456)	1445.51	2.62
Scenario 3_{v2}	1.586 (1.049-3.164)	0.865 (0.544-0.992)	0.888 (0.593-1.379)	-	1.538 (0.986-2.433)	0.333 (0.314-0.354)	1448.09	3.07
Scenario 3_{v3}	0.304 (0.293-0.315)	0.980 (0.900-0.999)	-	-	-	0.436 (0.412-0.464)	1462.71	1.95
Scenario 3_{v4}	0.321 (0.290-0.424)	0.830 (0.390-0.991)	-	-	0.436 (0.412-0.462)	0.434 (0.409-0.461)	1465.32	2.58

Supplementary Table 20: Parameter estimates for 1 cross-sections from Tanzania, Rombo District for PGP3 for the antibody acquisition models evaluated. The first value presented in each cell is the median parameter estimate and the values in the brackets below are the 95% credible intervals for the estimated

parameters (the 2.5% and 97.5% percentile of the posterior distribution). DIC of the best performing selected model is highlighted in red.

Scenario	$\propto_T$	γ	*r*	t_c	$\propto_{UG}$	*σ*	DIC	pD
Scenario 1_{v1}	0.297 (0.259-0.341)	-	0.141 (0.116-0.172)	-	-	0.453 (0.427-0.481)	1190.72	2.20
Scenario 2_{v1}	25.061 (20.814-28.617)	0.015 (0.013-0.019)	0.227 (0.266-0.290)	15.99 (14.49-17.31)	-	0.373 (0.354-0.397)	984.91	1.96
Scenario 2_{v2}	2.696 (1.597-3.593)	0.093 (0.069-0.159)	-	26.75 (23.09-29.28)	-	0.432 (0.411-0.459)	1142.43	2.46
Scenario 2_{v3}	1.815 (1.200-3.094)	0.694 (0.405-0.990)	1.089 (0.928-1.229)	45.46 (22.39-59.35)	1.452 (1.202-1.699)	0.336 (0.317-0.358)	859.78	-1.35
Scenario 2_{v4}	0.780 (0.134-0.941)	0.376 (0.213-0.941)	-	37.34 (28.12-56.72)	0.021 (0.001-0.053)	0.433 (0.409-0.459)	1146.65	2.22
Scenario 3_{v1}	0.387 (0.282-1.437)	0.725 (0.054-0.987)	0.151 (0.123-0.218)	-	-	0.452 (0.427-0.480)	1191.33	2.34
Scenario 3_{v2}	2.443 (1.563-2.777)	0.524 (0.358-0.842)	1.061 (0.834-1.450)	-	1.415 (1.083-1.917)	0.337 (0.318-0.358)	1194.19	2.44
Scenario 3_{v3}	0.273 (0.263-0.283)	0.948 (0.763-0.998)	-	-	-	0.453 (0.426-0.481)	1192.10	1.84
Scenario 3_{v4}	0.273 (0.217-0.287)	0.868 (0.551-0.994)	-	-	0.028 (0.008-0.050)	0.457(0.432-0.486)	1194.59	2.40

Supplementary Table 21: Parameter estimates for 1 cross-sections from Tanzania, Rombo District for CT694 for the antibody acquisition models evaluated. The first value presented in each cell is the median parameter estimate and the values in the brackets below are the 95% credible intervals for the estimated parameters (the 2.5% and 97.5% percentile of the posterior distribution). DIC of the best performing selected model is highlighted in red.

Scenario	$\propto_T$	γ	*r*	t_c	$\propto_{UG}$	*σ*	DIC	pD
Scenario 1_{v1}	80.213 (34.828-98.865)	-	1.395 (0.581-1.994)	-	-	1.988 (1.854-2.162)	4129.00	1.61
Scenario 1_{v2}	13.737 (10.994-17.034)	-	-	-	-	2.030 (1.885-2.199)	4142.51	1.39
Scenario 2_{v1}	601.769 (172.946-969.632)	0.592 (0.182-0.966)	6.042 (1.559-9.769)	29.712 (1.503-58.084)	-	1.986 (1.844-2.146)	4127.31	1.26
Scenario 2_{v2}	27.167 (10.321-135.378)	0.393 (0.078-0.965)	-	36.045 (11.243-59.042)	-	2.056 (1.921-2.203)	4768.12	1.43
Scenario 3_{v1}	18.098 (12.506-25.910)	0.914 (0.635-0.996)	0.208 (0.046-0.392)	-	-	2.016 (1.877-2.184)	4140.48	2.84
Scenario 3_{v3}	15.104 (11.51-17.855)	0.667 (0.066-0.985)	-	-	-	2.030 (1.888-2.193)	4143.80	1.56

Supplementary Table 22: Parameter estimates for 1 cross-sections from Fiji, iTaukei population for Pgp3 for the antibody acquisition models evaluated. The first value presented in each cell is the median parameter estimate and the values in the brackets below are the 95% credible intervals for the estimated parameters (the 2.5% and 97.5% percentile of the posterior distribution). DIC of the best performing selected model is highlighted in red.

Scenario	$\propto_T$	γ	*r*	t_c	$\propto_{UG}$	σ	DIC	pD
Scenario 1_{v1}	64.196 (4.459-97.021)	-	5.544 (0.268-9.751)	-	-	1.839 (1.515-2.275)	444.31	1.05
Scenario 1_{v2}	2.596 (1.637-4.146)	-	-	-	-	1.764 (1.472-2.170)	441.13	1.33
Scenario 2_{v1}	297.354 (38.086 – 968.119)	0.215 (0.027-0.894)	6.123 (0.481-9.794)	24.152 (1.700-59.095)	-	1.811 (1.515-2.210)	439.85	-3.89
Scenario 2_{v2}	32.711 (2.439-492.018)	0.078 (0.004-0.959)	-	37.153 (3.708-58.971)	-	1.782 (1.464-2.232)	440.97	1.22
Scenario 3_{v1}	6.442 (2.530-13.254)	0.599 (0.081-0.983)	0.260 (0.030-1.099)	-	-	1.770 (1.450-2.172)	442.49	1.70
Scenario 3_{v3}	2.788 (1.563-4.725)	0.508 (0.026-0.977)	-	-	-	1.763 (1.456-2.202)	441.44	1.38

Supplementary Table 23: Parameter estimates for 1 cross-sections from Fiji, Indo-Fijian population for Pgp3 for the antibody acquisition models evaluated. The first value presented in each cell is the median parameter estimate and the values in the brackets below are the 95% credible intervals for the estimated parameters (the 2.5% and 97.5% percentile of the posterior distribution). DIC of the best performing selected model is highlighted in red.

Scenario	$\propto_T$	γ	*r*	t_c	$\propto_{UG}$	σ	DIC	pD
Scenario 1_{v1}	58.688 (38.372-95.691)	-	0.130 (0.009-0.353)	-	-	1.649 (1.512-1.816)	3184.8	1.99
Scenario 1_{v2}	52.744 (42.132-66.560)	-	-	-	-	1.657 (1.510-1.828)	3183.2	1.43
Scenario 2_{v1}	220.228 (54.605 – 836.966)	0.318 (0.072-0.943)	0.136 (0.009-1.901)	31.207 (2.663-57.944)	-	1.660 (1.523-1.834)	3182.5	-0.50
Scenario 2_{v2}	182.883 (57.481-871.049)	0.289 (0.057-0.917)	-	29.359 (3.313-57.981)	-	1.646 (1.512-1.801)	3183.06	1.32
Scenario 3_{v1}	97.557 (45.092-306.088)	0.427 (0.012-0.973)	0.108 (0.006-0.443)	-	-	1.654 (1.503-1.832)	3185.01	1.90
Scenario 3_{v3}	57.059 (45.039-72.406)	0.455 (0.020-0.973)	-	-	-	1.652 (1.503-1.813)	3183.04	1.44

Supplementary Table 24: Parameter estimates for 1 cross-sections from Kiribati for Pgp3 for the antibody acquisition models evaluated. The first value presented in each cell is the median parameter estimate and the values in the brackets below are the 95% credible intervals for the estimate (the 2.5% and 97.5% percentile of the posterior distribution). DIC of the best performing selected model is highlighted in red.

Parameter estimates obtained from fitting to smaller sub-section of the data from Nepal

Model and data	PGP3-1-9 year olds	PGP3-1-16 year olds	PGP3-1-9 and 20-30 year olds	CT694-1-9 year olds	CT694-1-16 year olds	CT694-1-9 and 20-30 year olds
**Nepal-2 cross-sections**						
**Scenario 1_{v1}**						
**λ_T**	0.111 (0.049-0.270)	0.107 (0.045-0.299)	0.053 (0.041-0.074)	0.097 (0.054-0.226)	0.190 (0.051-0.339)	0.056 (0.044-0.079)
**ρ**	0.262 (0.038-0.596)	0.220 (0.048-0.651)	0.011 (0.0006-0.034)	0.206 (0.015-0.558)	0.412 (0.060-0.709)	0.009 (0.0004-0.032)
**Scenario 2_{v1}**						
**λ_T**	6.879 (1.657-9.863)	6.695 (0.481-9.824)	0.206 (0.142-0.326)	7.07 (1.91-9.83)	7.176 (1.092-9.890)	0.164

						(0.119-0.239)
**γ**	0.059 (0.017-0.147)	0.035 (0.010-0.062)	0.037 (0.015-0.071)	0.027 (0.005-0.090)	0.048 (0.021-0.081)	0.064 (0.038-0.101)
**ρ**	5.871 (1.130-9.585)	2.898 (0.181-5.226)	0.056 (0.033-0.087)	5.338 (1.458-9.226)	3.463 (0.509-5.939)	0.035 (0.020-0.058)
**t_c**	5.112 (0.608-9.727)	8.429 (1.074-14.965)	13.15 (11.61-15.81)	5.042 (1.458-9.226)	8.44 (0.893-14.70)	15.03 (13.12-15.94)
**Nepal-pre-MDA**						
**Scenario 1_{v1}**						
**λ_T**	1.269 (0.244-2.418)	1.700 (0.291-2.400)	0.294 (0.143-0.709)	0.809 (0.208-1.98)	1.286 (0.745-2.135)	0.183 (0.122-0.939)
**ρ**	0.740 (0.103-1.714)	0.727 (0.111-1.148)	0.094 (0.029-0.231)	0.608 (0.161-1.390)	0.610 (0.389-1.060)	0.044 (0.019-0.307)
**Scenario 2_{v1}**						
**λ_T**	6.436 (2.316-9.796)	8.994 (4.967-9.905)	5.438 (2.304-9.860)	3.124 (0.536-9.436)	2.511 (0.226-9.674)	5.646 (0.408-9.785)
**γ**	0.619 (0.172-0.977)	0.856 (0.488-0.993)	0.489 (0.149-0.974)	0.469 (0.067-0.963)	0.219 (0.017-0.937)	0.634 (0.124-0.979)
**ρ**	2.308 (0.964-3.902)	3.201 (1.764-4.208)	0.788 (0.374-1.449)	0.802 (0.132-3.329)	0.192 (0.044-0.908)	1.127 (0.037-1.758)
**t_c**	15.108 (0.717-29.122)	7.766 (0.249-15.659)	15.222 (0.677-29.246)	15.440 (0.725-29.264)	8.770 (0.802-15.537)	15.655 (0.730-29.220)
**Nepal-post-MDA**						
**Scenario 1_{v1}**						
**λ_T**	0.049 (0.010-0.162)	0.052 (0.008-0.120)	0.013 (0.009-0.017)	0.037 (0.007-0.129)	0.094 (0.048-0.167)	0.015 (0.011-0.020)
**ρ**	1.194 (0.837-1.789)	0.636 (0.033-1.186)	0.005 (0.0002-0.033)	0.599 (0.041-1.108)	0.939 (0.541-1.390)	0.004 (0.0001-0.029)
**Scenario 2_{v1}**						
**λ_T**	0.401 (0.021-2.964)	0.401 (0.021-2.964)	0.171 (0.028-1.268)	0.169 (0.014-2.156)	0.101 (0.013-1.581)	0.146 (0.035-0.924)
**γ**	0.194 (0.016-0.918)	0.139 (0.008-0.927)	0.034 (0.003-0.249)	0.109 (0.005-0.863)	0.167 (0.007-0.928)	0.043 (0.007-0.335)
**ρ**	2.174 (0.960-3.078)	0.584 (0.160-1.394)	0.029 (0.001-0.083)	0.420 (0.046-0.767)	0.270 (0.063-0.444)	0.013 (0.0007-0.047)
**t_c**	5.242 (0.497-9.749)	8.857 (1.289-15.564)	17.453 (6.593-27.12)	6.435 (0.800-9.775)	7.747 (1.144-15.574)	18.806 (8.635-26.972)

Supplementary Table 25. Parameter estimates from 1 or 2 cross-sections from Nepal from the sero-catalytic models with increasing amounts of data on different age groups provided for each fit. The first value presented in each cell is the median parameter estimate and the values in the brackets below are the 95% credible intervals for the estimate (the 2.5% and 97.5% percentile of the posterior distribution).

Supplementary Methods: Mathematical description of sero-catalytic and antibody acquisition models

Serocatalytic modelling methods

As outlined in (4) our approach was as follows. We accessed cross-sectional data on age-specific prevalence and titre data in arbitrary units from adults and children aged 1-90 available from 9 different epidemiological sites, these data were assumed to represent cumulative exposure to the antigens PGP3 and CT694. For data from Nepal, information on two cross-sections of the population was collected, once in the year 2000 and the second in 2014. For all other study sites, information was only available at one time point. For each model, we used t to denote time. t_{end} was the time of the most recent cross-sectional survey. t_{start} was the age of the oldest person at the first cross-sectional survey. The transmission period was therefore the period of time between t_{start} and t_{end}. We used the seroconversion rate for *Ct*, λ_T, as a marker for ocular *Ct* transmission intensity, and λ_{ug} as a marker for urogenital chlamydia transmission intensity. We considered 3 different patterns of change in transmission

intensity that may have occurred over time. For simplicity, we first considered a scenario where there was only one route to seroconversion:

Scenario 1: Constant transmission over time

$$\lambda\,(t) = \lambda_0$$

Scenario 2: An instantaneous drop in transmission at a given time point (t_c)

$$\lambda\,(t) = \begin{cases} \lambda_0 \; t < t_c \\ \lambda_c \; t \geq t_c \end{cases}$$

Scenario 3: A linear reduction in transmission over time

$$\lambda\,(t) = \lambda_0 + \frac{t}{t_{end}}\,(\lambda_c - \lambda_0)$$

Seronegative individuals become seropositive at a rate $\lambda\,(t)$ and seropositive individuals become seronegative at a rate ρ, therefore the proportion of seropositive individuals in a cross-section is described by the following differential equation:

$$\frac{dP}{dt} = \lambda\,(t)\,(1-P) - \rho P$$

This equation can be solved for the 3 different transmission assumptions to provide an estimate of the proportion of people who were seropositive at age a years.

Scenario 1: Constant transmission

$$P\,(a) = \frac{\lambda_0}{\lambda_0 + \rho}\,(1 - e^{-(\lambda_c + \rho)a})$$

Scenario 2: An instantaneous drop in transmission at a given time point (t_c years ago)

$$P\,(a) = \begin{cases} \frac{\lambda_c}{\lambda_c + \rho}\left(1 - e^{-(\lambda_c + \rho)a}\right) : \; a_c < 0 \\ \frac{\lambda_c}{\lambda_c + \rho} + \frac{(\lambda_0 - \lambda_c)\rho}{(\lambda_0 + \rho) + (\lambda_c + \rho)} e^{-(\lambda_c + \rho)(a - a_c)} - \frac{\lambda_0}{\lambda_0 + \rho} e^{-(\lambda_c + \rho)a} e^{-(\lambda_0 + \lambda_c)a_c} \; : \; 0 < a_c < a \\ \frac{\lambda_0}{\lambda_0 + \rho}\left(1 - e^{-(\lambda_0 + \rho)a}\right) : \; a_c > a \end{cases}$$

Where $a_c = a\text{-}t_c$ is the age of the individual at the time when the drop in transmission occurred.

Scenario 3 cannot be solved analytically and must be solved numerically.

The proportional drop in the rate of sero-conversion is defined as $\gamma = \frac{\lambda_c}{\lambda_0}$. Each sero-catalytic model was fitted to the age-dependent data on sero-positivity status using a binomial likelihood function. The likelihood that the model-predicted seropositive proportion $P(a_i)$ fits the proportion of seropositive individuals observed in the data k_i/N_i for all age groups in one cross-section was given by the binomial likelihood:

$$L(\theta|N_i, k_i) = \sum_{i=1}^{I} \binom{N_i}{k_i} P(a_i)^{k_i}(1 - P(a_i)^{N_i - k_i})$$

Where i is the age group, N_i the number of samples at age a_i, of which k_i test seropositive and θ is the parameter vector. For model 1, $\theta = (\lambda_0, \rho)$; for model 2, $\theta = (\lambda_0, \lambda_c, t_c, \rho)$; and for model 3, $\theta = (\lambda_0, \lambda_c, t_c)$. In order to fit two cross-sections of data, the product of the likelihoods of fitting each cross-section individually was taken to calculate the total likelihood. Markov Chain Monte Carlo simulation was performed for parameter estimation

assuming uniform priors and the binomial likelihood evaluated for each vector of parameters. Credible intervals for each parameter were estimated as the 2.5th and 97.5th percentile of the posterior distribution.

Modelling sero-prevalence data accounting for a secondary exposure to the antigen following the age of sexual debut.

We assume that once individuals reach the age of sexual debut, $a_{SD,}$ then they sero-convert at rate λ_{ug} due to exposure to urogenital *Ct*. The sero-conversion rate is then dependent on both age and time.

Scenario 1 with urogenital exposure:

$$\lambda(a,t)=\begin{cases}\lambda_0 & a<a_{SD}\\ \lambda_0+\lambda_{ug} & a\geq a_{SD}\end{cases}$$

Scenario 2 with urogenital exposure:

$$\lambda(a,t)=\begin{cases}\lambda_0 & a<a_{SD} & t<t_c\\ \lambda_0+\lambda_{ug} & a\geq a_{SD} & t<t_c\\ \lambda_c & a<a_{SD} & t\geq t_c\\ \lambda_c+\lambda_{ug} & a\geq a_{SD} & t\geq t_c\end{cases}$$

Scenario 3: A linear reduction in transmission from λ_0 at time t_0 to the the time of survey:

$$\lambda(a,t)=\begin{cases}\lambda_0+\dfrac{t}{t_0}(\lambda_c-\lambda_0) & a<a_{SD}\\ \lambda_{ug}+\lambda_0+\dfrac{t}{t_0}(\lambda_c-\lambda_0) & a\geq a_{SD}\end{cases}$$

Scenarios 1 and 2 can be solved analytically to provide expressions for $P(a)$: the proportion of individuals of age a who are sero-positive. Scenario 3 must be solved numerically.

Analytic solution for Scenario 1 with urogenital exposure:

Case 1: born; survey; sexual debut ($a < a_{SD}$)

$$P(a)=\frac{\lambda_0}{\lambda_0+\rho}\left(1-e^{-(\lambda_0+\rho)a}\right)$$

Case 2: born; sexual debut ($a \geq a_{SD}$); survey

$$P_{\mathrm{SD}}=\frac{\lambda_0}{\lambda_0+\rho}\left(1-e^{-(\lambda_0+\rho)a_{\mathrm{SD}}}\right)$$

$$P(a)=\left(1-P_{\mathrm{SD}}\right)\frac{\lambda_0+\lambda_{\mathrm{ug}}}{\lambda_0+\lambda_{\mathrm{ug}}+\rho}\left(1-e^{-\left(\lambda_0+\lambda_{\mathrm{ug}}+\rho\right)\left(a-a_{\mathrm{SD}}\right)}\right)+P_{\mathrm{SD}}\frac{\lambda_0+\lambda_{\mathrm{ug}}+\rho e^{-\left(\lambda_0+\lambda_{\mathrm{ug}}+\rho\right)\left(a-a_{\mathrm{SD}}\right)}}{\lambda_0+\lambda_{\mathrm{ug}}+\rho}$$

Scenario 2 with urogenital exposure:
Case 1: born; sexual debut ($a > a_{SD}$); transmission drops ($a - a_{SD} > t_c$); survey

$$P_{\text{SD}} = \frac{\lambda_0}{\lambda_0 + \rho}\left(1 - e^{-(\lambda_0+\rho)a_{\text{SD}}}\right)$$

$$P_{\text{tc}} = \left(1 - P_{\text{SD}}\right)\frac{\lambda_0 + \lambda_{\text{ug}}}{\lambda_0 + \lambda_{\text{ug}} + \rho}\left(1 - e^{-\left(\lambda_0+\lambda_{\text{ug}}+\rho\right)\left(a-a_{\text{SD}}-t_c\right)}\right) + P_{\text{SD}}\frac{\lambda_0 + \lambda_{\text{ug}} + \rho e^{-\left(\lambda_0+\lambda_{\text{ug}}+\rho\right)\left(a-a_{\text{SD}}-t_c\right)}}{\lambda_0 + \lambda_{\text{ug}} + \rho}$$

$$P(a) = \left(1 - P_{\text{tc}}\right)\frac{\lambda_c + \lambda_{\text{ug}}}{\lambda_c + \lambda_{\text{ug}} + \rho}\left(1 - e^{-\left(\lambda_c+\lambda_{\text{ug}}+\rho\right)\left(t_c\right)}\right) + P_{\text{tc}}\frac{\lambda_c + \lambda_{\text{ug}} + \rho e^{-\left(\lambda_c+\lambda_{\text{ug}}+\rho\right)\left(t_c\right)}}{\lambda_c + \lambda_{\text{ug}} + \rho}$$

Case 2: transmission drops ($a < t_c$); born; sexual debut ($a > a_{SD}$); survey

$$P_{\text{SD}} = \frac{\lambda_0}{\lambda_0 + \rho}\left(1 - e^{-(\lambda_0+\rho)a_{\text{SD}}}\right)$$

$$P(a) = \left(1 - P_{\text{SD}}\right)\frac{\lambda_0 + \lambda_{\text{ug}}}{\lambda_0 + \lambda_{\text{ug}} + \rho}\left(1 - e^{-\left(\lambda_0+\lambda_{\text{ug}}+\rho\right)\left(a-a_{\text{SD}}\right)}\right) + P_{\text{SD}}\frac{\lambda_0 + \lambda_{\text{ug}} + \rho e^{-\left(\lambda_0+\lambda_{\text{ug}}+\rho\right)\left(a-a_{\text{SD}}\right)}}{\lambda_0 + \lambda_{\text{ug}} + \rho}$$

Case 3: born; transmission drops ($a - a_{SD} < t_c$); sexual debut ($a > a_{SD}$); survey

$$P_{\text{tc}} = \frac{\lambda_0}{\lambda_0 + \rho}\left(1 - e^{-(\lambda_0+\rho)(a-t_c)}\right)$$

$$P_{\text{SD}} = \left(1 - P_{\text{tc}}\right)\frac{\lambda_c}{\lambda_c + \rho}\left(1 - e^{-(\lambda_c+\rho)(t_c-a+a_{\text{SD}})}\right) + P_{\text{tc}}\frac{\lambda_c + \rho e^{-(\lambda_c+\rho)(t_c-a+a_{\text{SD}})}}{\lambda_c + \rho}$$

$$P(a) = \left(1 - P_{\text{SD}}\right)\frac{\lambda_c + \lambda_{\text{ug}}}{\lambda_c + \lambda_{\text{ug}} + \rho}\left(1 - e^{-\left(\lambda_c+\lambda_{\text{ug}}+\rho\right)\left(a_{\text{SD}}\right)}\right) + P_{\text{SD}}\frac{\lambda_c + \lambda_{\text{ug}} + \rho e^{-\left(\lambda_c+\lambda_{\text{ug}}+\rho\right)\left(a_{\text{SD}}\right)}}{\lambda_c + \lambda_{\text{ug}} + \rho}$$

Case 4: born; transmission drops ($a > t_c$); survey; sexual debut

$$P_{\text{tc}} = \frac{\lambda_0}{\lambda_0 + \rho}\left(1 - e^{-(\lambda_0+\rho)(a-t_c)}\right)$$

$$P(a) = \left(1 - P_{\text{tc}}\right)\frac{\lambda_c}{\lambda_c + \rho}\left(1 - e^{-(\lambda_c+\rho)(t_c)}\right) + P_{\text{tc}}\frac{\lambda_c + \rho e^{-(\lambda_c+\rho)(t_c)}}{\lambda_c + \rho}$$

Case 5: born; survey; sexual debut ($a < a_{SD}$); transmission drops ($t_c < 0$);

$$P(a) = \frac{\lambda_0}{\lambda_0 + \rho}\left(1 - e^{-(\lambda_0+\rho)a}\right)$$

Case 6: transmission drops ($a < t_c$); born; survey; sexual debut ($a < a_{SD}$);

$$P(a)=\frac{\lambda_c}{\lambda_c+\rho}\left(1-e^{-(\lambda_c+\rho)a}\right)$$

Case 7: transmission drops ($a < t_c$); born; sexual debut ($a > a_{SD}$); survey

$$P_{\text{SD}}=\frac{\lambda_c}{\lambda_c+\rho}\left(1-e^{-(\lambda_c+\rho)a_{\text{SD}}}\right)$$

$$P(a)=\left(1-P_{\text{SD}}\right)\frac{\lambda_c+\lambda_{\text{ug}}}{\lambda_c+\lambda_{\text{ug}}+\rho}\left(1-e^{-(\lambda_c+\lambda_{\text{ug}}+\rho)(a-a_{\text{SD}})}\right)+P_{\text{SD}}\frac{\lambda_c+\lambda_{\text{ug}}+\rho e^{-(\lambda_c+\lambda_{\text{ug}}+\rho)(a-a_{\text{SD}})}}{\lambda_c+\lambda_{\text{ug}}+\rho}$$

Antibody acquisition modelling methods

Antibody acquisition models use continuous antibody levels instead of dichotomizing individuals as seropositive or seronegative as seroprevalence models do. A comparison of the two approaches can be found in (1). We assume the rate at which antibody levels are acquired is α and is subscripted with T or UG to refer to antibodies acquired due to ocular or urogenital exposure. This rate was used as a marker for transmission intensity. If an individual's antibody levels were boosted at a rate $\alpha(t)$ and decayed at a rate r, then antibody levels can be described by the following differential equation:

$$\frac{dA}{dt}=\alpha(t)-rA$$

We assume that changes in $\alpha(t)$ correspond to changes in transmission intensity, as described for the sero-prevalence models.

Scenario 1: Constant transmission over time

$$A(a)=\frac{\alpha_0}{r}\left(1-e^{-ra}\right)$$

Scenario 2: An instantaneous drop in transmission at a given time point (t_c)

$$A(a)=\begin{cases}\frac{\alpha_c}{r}\left(1-e^{-ra}\right): a_c<0\\ \frac{\alpha_0}{r}e^{-ra}\left(e^{ra_c}-1\right)+\frac{\alpha_c}{r}\left(1-e^{-r(a-\alpha_c)}\right): 0<a_c<a\\ \frac{\alpha_0}{r}\left(1-e^{-ra}\right): a_c>a\end{cases}$$

Where a_c is the age of the child at the time when transmission dropped.

Scenario 3: A linear reduction in transmission from α_0 at time t_0 to α_c at the time of survey:

$$A(a)=\left(\alpha_c+\left(\alpha_0-\alpha_c\right)\frac{a}{t_0}+\frac{\alpha_0-\alpha_c}{rt_0}\right)\frac{1-e^{-ra}}{r}-\frac{\alpha_0-\alpha_c}{r}\frac{a}{t_0}$$

The magnitude of reduction in the rate of antibody acquisition was calculated as $\gamma=\frac{\alpha_c}{\alpha_0}$. The antibody acquisition model was fitted to age-dependent antibody level data, with one or two cross-sections depending on the dataset, assuming the geometric mean antibody level at age a is $A(a)$ and that antibody levels are log-normally distributed in the cohort with the standard deviation on the log scale σ. To calculate the likelihood of a given parameter set for the antibody acquisition model, for an individual k of age a_k with the antibody level x_k, the likelihood that the model predicted geometric antibody level $A(a_k)$ fits the data for a given individual is given by:

$$L(\theta|x_k, a_k) = \frac{1}{x_k \sigma \sqrt{2\pi}} e^{\frac{e^{(\log(x_k) - \log(A(a_k)))^2}}{2\sigma^2}}$$

where θ is the parameter vector. For model 1, this is $\theta = (\alpha_0, \sigma, r)$; for model 2, this is $\theta = (\alpha_0, \alpha_c, \sigma, r, t_c)$ and for model 3, $\theta = (\alpha_0, \alpha_c, \sigma, r)$. The likelihood of fitting the model to data from two cross-sections was the product of the likelihoods from both cross-sections when they were individually summed across all individuals in each of the separate cross-sections.

Modelling antibody level data accounting for a secondary exposure to the antigen following the age of sexual debut.

We assume that once individuals reach the age of sexual debut, a_{SD}, then they start acquiring antibodies at rate α_{ug}, due to exposure to urogenital *Ct*. The antibody acquisition rate is then dependent on both age and time.

Scenario 1 with urogenital exposure:

$$\alpha(a,t) = \begin{cases} \alpha_0 & a < a_{SD} \\ \alpha_0 + \alpha_{ug} & a \geq a_{SD} \end{cases}$$

Scenario 2 with urogenital exposure:

$$\alpha(a,t) = \begin{cases} \alpha_0 & a < a_{SD} & t < t_c \\ \alpha_0 + \alpha_{ug} & a \geq a_{SD} & t < t_c \\ \alpha_c & a < a_{SD} & t \geq t_c \\ \alpha_c + \alpha_{ug} & a \geq a_{SD} & t \geq t_c \end{cases}$$

Scenario 3: A linear reduction in transmission over time:

$$\alpha(a,t) = \begin{cases} \alpha_0 + \frac{t}{t_0}(\alpha_c - \alpha_0) & a < a_{SD} \\ \alpha_{ug} + \alpha_0 + \frac{t}{t_0}(\alpha_c - \alpha_0) & a \geq a_{SD} \end{cases}$$

These models can be solved analytically to provide expressions for $A(a)$: the geometric mean titre at age a.

Analytic solution for Scenario 1 with urogenital exposure:

Case 1: born; survey; sexual debut ($a < a_{SD}$)

$$A(a) = \frac{\alpha_0}{r}\left(1 - e^{-ra}\right)$$

Case 2: born; sexual debut ($a \geq a_{SD}$); survey

$$A_{SD} = \frac{\alpha_0}{r}\left(1 - e^{-ra_{SD}}\right)$$

$$A(a) = A_{SD} e^{-r(a - a_{SD})} + \frac{\alpha_0 + \alpha_{ug}}{r}\left(1 - e^{-r(a - a_{SD})}\right)$$

Analytic solution for Scenario 2 with urogenital exposure:

Case 1: born; sexual debut ($a > a_{SD}$); transmission drops ($a - a_{SD} > t_c$); survey

$$A_{\text{SD}} = \frac{\alpha_0}{r}\left(1 - e^{-ra_{\text{SD}}}\right)$$

$$A_{\text{tc}} = A_{\text{SD}} e^{-r\left(a - a_{\text{SD}} - t_c\right)} + \frac{\alpha_0 + \alpha_{\text{ug}}}{r}\left(1 - e^{-r\left(a - a_{\text{SD}} - t_c\right)}\right)$$

$$A(a) = A_{\text{tc}} e^{-r\left(t_c\right)} + \frac{\alpha_c + \alpha_{\text{ug}}}{r}\left(1 - e^{-r\left(t_c\right)}\right)$$

Case 2: transmission drops ($a < t_c$); born; sexual debut ($a > a_{SD}$); survey

$$A_{\text{SD}} = \frac{\alpha_0}{r}\left(1 - e^{-ra_{\text{SD}}}\right)$$

$$A(a) = A_{\text{SD}} e^{-r\left(a - a_{\text{SD}}\right)} + \frac{\alpha_0 + \alpha_{\text{ug}}}{r}\left(1 - e^{-r\left(a - a_{\text{SD}}\right)}\right)$$

Case 3: born; transmission drops ($a - a_{SD} < t_c$); sexual debut ($a > a_{SD}$); survey

$$A_{\text{tc}} = \frac{\alpha_0}{r}\left(1 - e^{-r\left(a - t_c\right)}\right)$$

$$A_{\text{SD}} = A_{\text{tc}} e^{-r\left(t_c - a + a_{\text{SD}}\right)} + \frac{\alpha_c}{r}\left(1 - e^{-r\left(t_c - a + a_{\text{SD}}\right)}\right)$$

$$A(a) = A_{\text{SD}} e^{-r\left(a_{\text{SD}}\right)} + \frac{\alpha_c + \alpha_{\text{ug}}}{r}\left(1 - e^{-r\left(a_{\text{SD}}\right)}\right)$$

Case 4: born; transmission drops ($a > t_c$); survey; sexual debut

$$A_{\text{tc}} = \frac{\alpha_0}{r}\left(1 - e^{-r\left(a - t_c\right)}\right)$$

$$A(a) = A_{\text{tc}} e^{-r\left(t_c\right)} + \frac{\alpha_c}{r}\left(1 - e^{-r\left(t_c\right)}\right)$$

Case 5: born; survey; sexual debut ($a < a_{SD}$); transmission drops ($t_c < 0$);

$$A(a) = \frac{\alpha_0}{r}\left(1 - e^{-ra}\right)$$

Case 6: transmission drops ($a < t_c$); born; survey; sexual debut ($a < a_{SD}$);

$$A(a) = \frac{\alpha_c}{r}\left(1 - e^{-ra}\right)$$

Case 7: transmission drops ($a < t_c$); born; sexual debut ($a > a_{SD}$); survey

$$A_{\text{SD}} = \frac{\alpha_c}{r}\left(1 - e^{-ra_{\text{SD}}}\right)$$

$$A(a) = A_{\text{SD}} e^{-r\left(a - a_{\text{SD}}\right)} + \frac{\alpha_c + \alpha_{\text{ug}}}{r}\left(1 - e^{-r\left(a - a_{\text{SD}}\right)}\right)$$

Analytic solution Scenario 3: A linear reduction in transmission over time:

Case 1: born; survey; sexual debut ($a < a_{SD}$)

$$A(a)=\left(\alpha_c+(\alpha_0-\alpha_c)\frac{a}{t_0}+\frac{\alpha_0-\alpha_c}{rt_0}\right)\frac{1-e^{-ra}}{r}-\frac{\alpha_0-\alpha_c}{rt_0}a$$

Case 2: born; sexual debut ($a > a_{SD}$); survey

$$A_{\mathrm{SD}}=\left(\alpha_c+(\alpha_0-\alpha_c)\frac{a_{\mathrm{SD}}}{t_0}+\frac{\alpha_0-\alpha_c}{rt_0}\right)\frac{1-e^{-ra_{\mathrm{SD}}}}{r}-\frac{\alpha_0-\alpha_c}{rt_0}a_{\mathrm{SD}}$$

$$A(a)=A_{\mathrm{SD}}e^{-r(a-a_{\mathrm{SD}})}+\left(\alpha_c+(\alpha_0-\alpha_c)\frac{t_0-a_{\mathrm{SD}}}{t_0}\frac{a-a_{\mathrm{SD}}}{a_{\mathrm{SD}}}+\frac{\alpha_0-\alpha_c}{ra_{\mathrm{SD}}}\frac{t_0-a_{\mathrm{SD}}}{t_0}\right)\frac{1-e^{-r(a-a_{\mathrm{SD}})}}{r}$$

$$-\frac{\alpha_0-\alpha_c}{ra_{\mathrm{SD}}}\frac{t_0-a_{\mathrm{SD}}}{t_0}(a-a_{\mathrm{SD}})$$

Model fitting and comparison

For each model, we ran at least 50,000 Markov Chain Monte Carlo (MCMC) iterations. The first 20% of MCMC iterations were assumed to be the burn in period and were discarded from the posterior distribution analysis. 2 MCMC chains were run for each model. We calculated the effective sample size for the posterior distributions of each model to ensure that they were >350 for each parameter estimated. Chain convergence was assessed ensuring that Rhat was >1.01 for each parameter for each model. Parameter estimation by MCMC was performed assuming uniform priors for each parameter. Ranges for each of the prior distributions were consistent across all models and study sites evaluated, and were as follows for the sero-prevalence models: λ_t ~ U(0,10), t_c ~ U(0,90) (upper range was set to the maximum age of an individual within the dataset), ρ ~ U(0,10), λ_{ug} ~ U(0,10) and γ ~ U(0,1). For the antibody acquisition model: $\alpha_t \sim U(0,1000), \sigma \sim U(0,1)$, $r \sim U(0,10), t_c \sim U(0,90)$(upper range was set to the maximum age of an individual within the dataset), $\alpha_{ug} \sim U(0,1000)$. All models were statistically compared using the Deviance Information Criteria (DIC) and were calculated as previously described in (5). We also calculated the pD, a measure of complexity and hence an estimate of the number of effective parameters. For the majority of study sites only one-cross section was available, which can make it challenging to distinguish between different competing hypotheses about changes in transmission over time. Therefore, in order to select the best performing model, we evaluated both the value of the DIC and AIC (a more consistent measure of model fit to the data) and also selected the most appropriate model based of the epidemiological history of the setting and our understanding of interventions that have been conducted within the population under study. For settings in which we were aware of a moderate/high incidence urogenital infection, or had data derived from the presumed (on the basis of age) on the sexually-active population (Tanzania, The Gambia, Solomon Islands), we only considered models that accounted for a secondary exposure to *Ct* antigens through urogenital *Ct*. Equally, we did not consider models that assumed a fixed change point in transmission as appropriate unless an intensive intervention programme was known to have occurred at a fixed point in time within the population. Models that were excluded based on our understanding of the epidemiology of a given setting or because of parameter estimation challenges were highlighted in green and yellow, respectively. Models shaded in green were excluded because they did not include exposure to urogenital *Ct*. Models shaded in orange were excluded for attempting to freely fit the sero-reversion rate (SRR). As we were interested in making comparisons of sero-conversion rates (SCR) between sites, it was highly beneficial to fix SRR: the difficulty of simultaneously estimating both the SCR and SRR from a single cross-sectional data set is well established. By fixing SRR with the value from Nepal, we allowed comparison of SCR between sites. We note that we did not discount models that fixed the rate of antibody decay in the antibody acquisition models, as the rates of acquisition and decay were not comparable across the Luminex and ELISA platforms.

Model types evaluated

We evaluated up to 10 different models for each study site, and 6 for sites for which data were only available for individuals <16 years of age. The methods outlined below have previously been applied and reported (1). The scenarios we evaluated were as follows:

Scenario 1_{v1} = A constant level of trachoma transmission in the community over time.
Scenario 1_{v2} = A constant level of trachoma transmission in the community (λ_T), but assuming the sero-reversion rate (ρ) or rate of antibody decay (r) to be fixed, using a previous estimate.
Scenario 2_{v1} = An instantaneous drop in the level of transmission (λ_T) at an estimated point in time ($\mathbf{t_c}$).
Scenario 2_{v2} = An instantaneous drop in the level of transmission (λ_T) at an estimated point in time ($\mathbf{t_c}$), but assuming the sero-reversion rate (ρ) or rate of antibody decay (r) to be fixed, using a previous estimate.
Scenario 2_{v3} = An instantaneous drop in the level of transmission (λ_T) at an estimated point in time ($\mathbf{t_c}$), but also accounting for secondary exposure to *Ct* antigens due to transmission of urogenital *Ct* ($\boldsymbol{\lambda_{UG}}$) after the age of sexual debut.
Scenario 2_{v4} = An instantaneous drop in the level of transmission (λ_T) at an estimated point in time ($\mathbf{t_c}$), but accounting for secondary exposure to *Ct* antigens due to transmission of urogenital *Ct* ($\boldsymbol{\lambda_{UG}}$) after the age of sexual debut, and assuming the sero-reversion rate (ρ) or rate of antibody decay (r) to be fixed, using a previous estimate.
Scenario 3_{v1} = A continuous drop in the level of transmission (λ_T) over time.
Scenario 3_{v2} = A continuous drop in the level of transmission (λ_T) over time, but also accounting for secondary exposure to *Ct* antigens due to transmission of urogenital *Ct* ($\boldsymbol{\lambda_{UG}}$) after the age of sexual debut.
Scenario 3_{v3} = A continuous drop in the level of transmission (λ_T) over time, but assuming the sero-reversion rate (ρ) or rate of antibody decay (r) to be fixed, using a previous estimate.
Scenario 3_{v4} = A continuous drop in the level of transmission (λ_T) over time, but also accounting for secondary exposure to *Ct* antigens due to transmission of urogenital *Ct* ($\boldsymbol{\lambda_{UG}}$) after the age of sexual debut, and assuming the sero-reversion rate (ρ) or rate of antibody decay (r) to be fixed, using a previous estimate.

Supplementary Discussion: Results from the antibody acquisition model and discussion on the impact of age-specific sampling for parameter estimation.

Results and fits of each antibody acquisition model to each dataset

For data from LRR, URR from The Gambia and Temuto and Rennell & Bellona provinces from the Solomon Islands, OD data were converted into arbitrary units (AUs). The AA model suggested that scenario 2_{v3} (a step-wise reduction in transmission), which had a free rr and also accounted for exposure to urogenital *Ct* was most appropriate for all four populations (Supplementary Figures 13-16, Supplementary Tables 15-18), in contrast to the SP model that selected a continuous decline. For all regions, t_c was imprecise and was estimated to be 3–59 years ago. As with the SP model, the estimated value of γ for all regions was also relatively unidentifiable, however precision of the estimate with the AA model was slightly better than with the SP model. The rate of α_T was higher than α_{UG} for LRR and URR, possibly reflecting a lower overall prevalence of urogenital *Ct* infection in these communities, by contrast α_{UG} was higher than α_T in the Solomon Islands, reflecting the high reported prevalence of urogenital *Ct* infection in these communities.

For Rombo, the AA modelling suggested scenario 2_{v3} (a single step-wise reduction in transmission, which had a free rr (rate of antibody decay) and also accounted for exposure to urogenital *Ct*) was most appropriate (Supplementary Figures 15 & 16, Supplementary Tables 17 & 18). Models that accounted for exposure to urogenital infection at sexual debut performed statistically better than those that did not. As with the SP model, t_c was also relatively unidentifiable. However, in contrast to the SP models, AA models where the rr was fixed performed less well than when it was a free parameter. Estimates of α_T and α_{UG} were markedly higher than estimated for Nepal, but the rr was 0.88 years (CrI: 0.75–1.05 years), possibly reflecting a trade-off in the estimation of α and rr. This pattern was observed for both antigens.

For populations in which data were only available for individuals <16 years of age (Kiribati and Fiji) the selected transmission scenario for each region was the same was the AA model as with the SP model. Analysis of Fiji data suggested that there was a constant level of transmission in the community. In both ethnic groups it was estimated that antibodies decayed and were acquired rapidly (Supplementary Tables 21 & 22). It was estimated that transmission scenario 3 (constant decline) was most appropriate for Kiribati. However, all models were almost statistically indistinguishable from one another, highlighting the difficulties associated with estimating parameters from a single cross-section collected from only the youngest age groups (Supplementary Table 23).

How does age-specific sampling impact parameter estimation?

Two cross-sectional surveys were available for Nepal, so it was possible to compare the parameters estimated from one vs two cross-sections in this setting. Identifiability of parameters can become challenging when only one cross-sectional survey has been conducted from a small age group. Precision of parameter estimation improved when samples were included from individuals >9 years old by fitting together the 2 cross-sections from Nepal. As the age range of individuals sampled increased (through the inclusion of adults), the estimated duration of ρ increased, but the estimated value of λ_T decreased (Supplementary Table 25). This reflects the lower overall mean population-level exposure to ocular *Ct* infection, but higher intensity and long-lived antibody responses (as seen in adults). The minimum age range that needed to be included in order to re-estimate the value of the parameters estimated from the full dataset was 1–9 and 20–30-year-olds. Observations were consistent for both antigens (Supplementary Table 25).

Evaluating the fits of the two cross-sections individually the median estimated λ_T for the pre-MDA cross-section was higher than the post-MDA cross-section. Increasing the range of age-specific data in the post-MDA data resulted in a lower median estimated λ_T for model 2 in comparison to when only data on younger individuals were available (0.052 vs 0.013, Supplementary Table 25). This did not hold true in the pre-MDA setting. A reduction in the median λ_T was seen consistently for both datasets with an increase in the age range evaluated for model 1 (1.26, 1.70, 0.29, for the pre-MDA data). Assessing the post-MDA cross-section t_c was not identifiable until older age groups were added (Supplementary Table 25), suggesting that if only 1–9-year-olds were sampled this would impact our understanding of historical patterns of transmission within the population. Additionally if we wish to accurately estimate parameters such as the SRR we need to select a model which accurately represents the transmission history within the community.

For a single cross-section, increasing the breadth of age groups evaluated slightly increased the precision of the estimate, but estimates were never as precise as when two cross-sections were fitted simultaneously (Supplementary Table 25). Parameter estimates differed when comparing the fits from one vs two cross-sections. Median parameter estimates for model 1 obtained from fitting both cross-sections simultaneously were more similar to the estimates from the post-MDA data fits. For data from individuals aged 1-9 years derived from the 2 cross-sections, we estimated λ_T as 0.11 (CrI: 0.04-0.27); λ_T was estimated to be 0.04 (CrI: 0.01-0.16) (Supplementary Table 25) from the post-MDA cross-section. However, for model 2, parameter estimates from the two cross-sections when looking at the first 2 age groups sampled were comparable to those estimated from the pre-MDA cross-section alone; 6.87 (CrI: 1.65-9.86) vs 6.43 (CrI: 2.31-9.79) (Supplementary Table 25). Once older age groups were included, estimates for each of the parameters were more similar to those from the post-MDA data that included the same age groups (Supplementary Table 25).

With 2 cross-sectional surveys, sampling individuals aged 1-9 years and 20-30 years produced similar parameter estimates to when data from all individuals aged 1-90 years from both cross-sections were fitted simultaneously. This suggests that although more than one cross-section should be taken in order to accurately estimate epidemiological parameters it is unlikely that multiple surveys would need to be conducted across the entire population; sampling from the two aforementioned age groups would be sufficient. Through having at least two cross-sections from the same population can ensure that both the SRR and SCR are identifiable parameters, as previously highlighted in the main text of this article, in the presence of only one cross-section parameters can be unidentifiable or trade-off with one another, making their accurate estimation challenging. Accurate estimation of these parameters is important if we are to understand long-term transmission and antibody dynamics within populations following cessation of interventions.

Supplementary References

1. R: A language and environment for statistical computing (R Foundation for statistical computing, Vienna, Austria)

2. South, Andy 2011 rworldmap: A New R package for Mapping Global Data. The R Journal Vol. 3/1 : 35-43.

3. Gelman A, Rubin DB. Inference from iterative simulation using multiple sequences. Statist Sci. 1992:457-72.

4. Yman V, White MT, Rono J, Arca B, Osier FH, Troye-Blomberg M, et al. Antibody acquisition models: A new tool for serological surveillance of malaria transmission intensity. Sci Rep. 2016;6:19472.

5. Spiegelhalter DJ, Best NG, Carlin BP, Van Der Linde A. Bayesian measures of model complexity and fit. *J R Statist Soc B*. 2002;64(4):583-639.
